# Supplementary material for: Identifying causal variants by fine mapping across multiple studies
Source: PLoS Genet. 2021 Sep 20;17(9):e1009733. doi: 10.1371/journal.pgen.1009733 (PMC8491908; doi:10.1371/journal.pgen.1009733)
Supplement: S1 Text — (PDF) [file pgen.1009733.s001.pdf]

Supplementary Note: Identifying Causal Variants by Fine Mapping Across Multiple Studies

Nathan LaPierre[1,†] Kodi Taraszka [1,†] Helen Huang[2] Rosemary He[3]
Farhad Hormozdiari[6] Eleazar Eskin[1,4,5,*]

[1]Department of Computer Science, University of California, Los Angeles, California, 90095, United States
[2]Bioinformatics Interdepartmental Program, Los Angeles, California, 90095, United States
[3]Department of Mathematics, University of California, Los Angeles, Los Angeles, California, 90095, United States
[4]Department of Human Genetics, University of California, Los Angeles, California, 90095, United States
[5]Department of Computational Medicine, University of California, Los Angeles, California, 90095, United States
[6]Harvard T.H. Chan School of Public Health, Boston, Massachusetts, 02115, United States
† These authors contributed equally
∗ Email corresponding author at: eeskin@cs.ucla.edu

1 Additional simulations

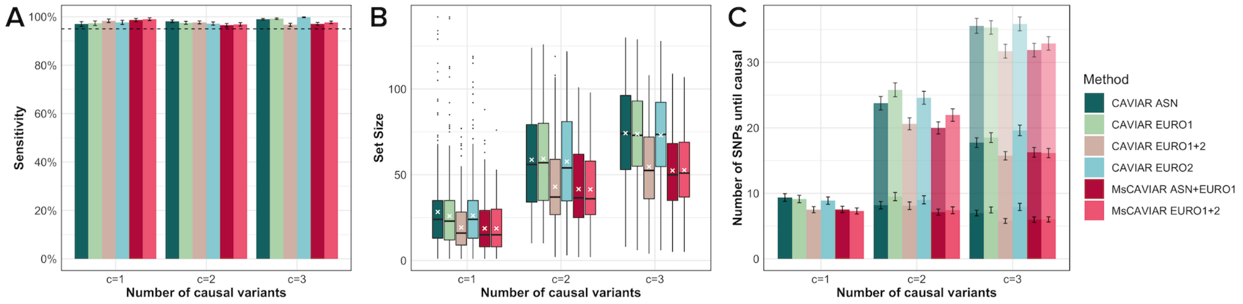

Fig A. Comparison of the impact of effective sample size increase to modeling heterogeneity. We simulate summary statistics with c=1, c=2, or c=3 causal variants implanted in 3 loci and 5 heritability levels with 20 replicates each. This is done for 2 different European populations and one Asian sample, all with 9,000 individuals in order to compare the impact leveraging differing LD to the effective sample size increase of meta-analysis. (A) Bar graph displaying the sensitivity of the methods (B) Box plots showing the set sizes returned by the methods. The lines inside the boxes represent the median while the white crosses inside the boxes represent the mean. (C) Bar graph showing the average number of SNPs taken in descending order of posterior inclusion probability (PIP) until 1, 2, or 3 causal SNPs are identified. Stacked bars represent increasing numbers of causal SNPs identified, until the true number of causal SNPs (x-axis) are identified.

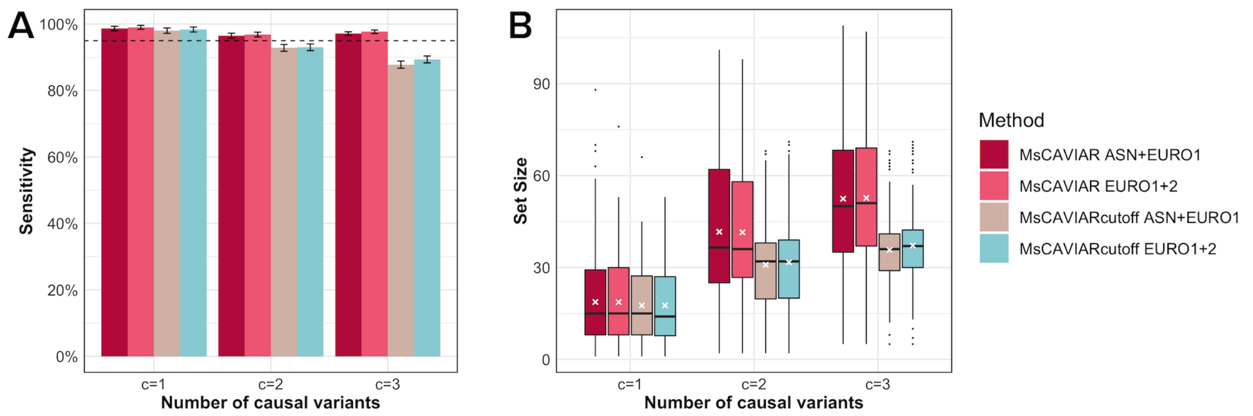

Fig B. Comparison of sensitivity and set sizes of MsCAVIAR to itself with a PIP cutoff. We simulated $c \in \{1, 2, 3\}$ causal variants averaging over 20 replications of 3 loci with 5 levels of heritability each. We compare the performance of MsCAVAIR to MsCAVIAR plus a posterior inclusion probability (PIP) threshold of 1%, where any SNPs with a PIP less than 1% is removed from the causal set. (A) Bar graph displaying the sensitivity of the methods. The dashed line reflects the expected posterior probability of recovering all causal SNPs; methods that reach this threshold are considered "well-calibrated". (B) Box plots showing the set sizes returned by the methods.

We performed additional simulations on the data described in the main text ("MsCAVIAR improves fine mapping resolution in a simulation study") including several other approaches. We sampled another population of 9,000 unrelated White British individuals. We then ran MsCAVIAR on the two British populations. We also ran fastGWA on the two European populations to generate stronger summary statistics, and then applied CAVIAR to those statistics (Fig A). We evaluated the effect of applying a "cutoff parameter" to MsCAVIAR's results, in which SNPs with less than 1% posterior probability of being causal were excluded from the causal set (Fig B). We ran simulations in which the causal effect sizes of the SNPs were equal across populations, in order to investigate whether lack of effect size heterogeneity impacted the results (Fig C). Finally, we ran SuSiE [1], a recent fine mapping method, on the Asian population and on the first British population (Fig D). We caution that SuSiE employs a different approach to fine mapping. SuSiE's credible sets differ from the causal sets of the other methods in that SuSiE does not attempt to capture all causal SNPs, so the sensitivity calibration is not directly comparable to the other methods. We focus on the rest of the methods in the following paragraphs, and then return to SuSiE.

MsCAVIAR run on the two British populations performed almost identically to MsCAVIAR run on the Asian and British population. Additionally, CAVIAR run on the two British populations yielded causal set sizes only marginally larger than MsCAVIAR's. Thus, in our simulations, combined population size was a larger factor than ethnic differences in LD. We caution that this may not always be the case in real data.

When a cutoff parameter was applied to MsCAVIAR, predictably, both the causal set size and recall were reduced. This reduction was proportional to the number of causal SNPs. With one causal SNP, the mean set size was reduced from 18.7 to 17.6 and the recall was reduced from 98.6% to 98%. With two causal SNPs, the mean set size was reduced from 41.6 to 30.9 and the recall was reduced from 96.5% to 92.8%. With three causal SNPs, the mean set size was reduced from 52.4 to 35.7 and the recall was reduced from 97.1% to 87.8%. The cutoff parameter, therefore, can be

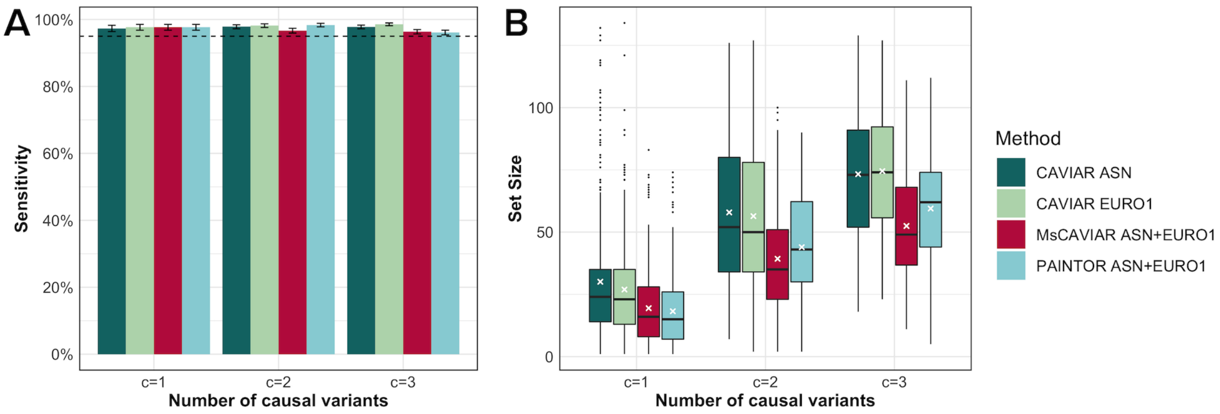

Fig C. Comparison of sensitivity and set sizes using simulated data with equal effect sizes. We simulated $c \in \{1, 2, 3\}$ causal variants averaging over 20 replications of 3 loci with 5 levels of heritability each. (A) Bar graph displaying the sensitivity of the methods. The dashed line reflects the expected posterior probability of recovering all causal SNPs; methods that reach this threshold are considered "well-calibrated". (B) Box plots showing the set sizes returned by the methods.

used to limit the causal set size, with the drawback of potentially reducing recall.

When we performed our original simulations except with effect sizes fixed across populations (e.g. no heterogeneity), the results were fairly similar to our main text results. This indicates that, in our simulations, MsCAVIAR's improved performance relative to PAINTOR is not mostly due to explicit modeling of heterogeneity.

SuSiE [1] takes a different approach to fine mapping from the other methods. Instead of returning a causal set, SuSiE returns (potentially multiple) credible sets for a locus, each of which is expected to contain at least one causal SNP. The goal of SuSiE is not to capture all causal variants in a locus, but to return one or more minimal size credible sets, each of which has ρ probability of containing at least one true causal effect. This explains why SuSiE is not well-calibrated according to our causal set definition, which expects all casual variants to be captured with probability ρ^*, when there is more than one causal SNP.

It is worth noting, however, that SuSiE's credible set is equivalent to the causal set (as defined by the other methods) when the methods assume that there is only one causal SNP in a locus. In this case, SuSiE applied to our Asian population had a recall of 91% and an average set size of 21.68, while SuSiE applied to the first of our British populations had a recall of 92.3% and an average set size of 20.6. Thus, SuSiE's returned set sizes were smaller than those of CAVIAR applied to individual populations but larger than MsCAVIAR's or PAINTOR's sets.

2 MsCAVIAR improves fine mapping resolution in a simulation study

Here, we describe a previous set of simulations that were removed from the main text during the revision process when the simulations now in the main text were developed. We have included them because they can complement our main simulations.

We used two regions from the 1000 Genomes project [2] to generate LD matrices for the SNPs

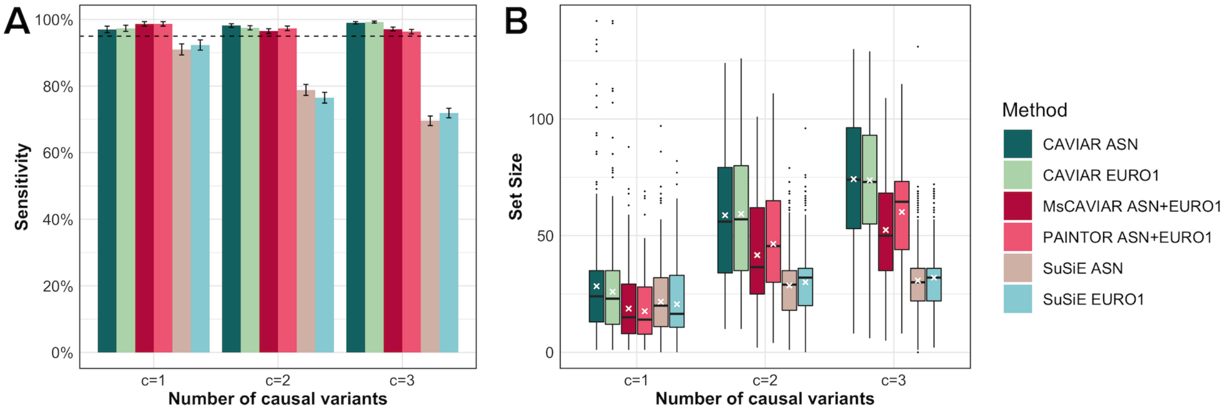

Fig D. Comparison of sensitivity, set sizes and precision in simulated data (w/ SuSiE). Comparison of the methods with c=1, c=2, or c=3 causal variants implanted. Results are averaged over three loci and 5 levels of heritability. The simulations are identical to those in the Fig 2 but we now include SuSiE, a fundamentally different method, in the comparison. SuSiE's credible sets differ from the causal sets of the other methods in that SuSiE does not attempt to capture all causal SNPs, so the sensitivity calibration is not directly comparable to the other methods. (A) Bar graphs displaying the sensitivity of the methods. The dashed line reflects the expected posterior probability of recovering all causal SNPs; methods that reach this threshold are considered "well-calibrated". (B) Box plots showing the set sizes returned by the methods. The boxes represent the interquartile range of causal set sizes identified by each tool, the lines inside the boxes represent the median while the white crosses inside the boxes represent the mean.

at that locus for both European and East Asian populations. Out of these loci, we selected one region with relatively low LD, where 20% of the SNP pairs have LD equal to or higher than 0.5, and one region with relatively high LD, where 80% of the SNP pairs have LD equal to or higher than 0.5 (Fig E, LD matrices). These represent easier and more difficult scenarios, respectively, for fine mapping, since LD makes signals more difficult to distinguish. We pruned groups of SNPs that were in perfect LD in one or more of the populations, leaving one SNP for each. After pruning, the low LD matrix contained 48 SNPs and the high LD matrix contained 38 SNPs.

Using these LD matrices, we implanted causal SNPs and simulated their non-centrality parameters. In each simulation, we implanted either 1, 2, or 3 causal SNPs. Each causal SNP's true non-centrality parameter Λ was drawn according to $\mathcal{N}(5.2, 0.125^2)$. We then drew the non-centrality parameter Λ_i for each study i according to $\Lambda_i \sim \mathcal{N}(\Lambda, 0.5)$, and subsequently the summary statistics S_i for each study i according to $S_i \sim \mathcal{N}(\Lambda_i \Sigma_i, \Sigma_i)$. For each number of causal SNPs, we performed 1000 replicate simulations (e.g. re-drawing the causal SNP non-centrality parameters and re-picking the causal SNPs).

Using this data, we compared MsCAVIAR to the trans-ethnic mode of PAINTOR [3] and to CAVIAR [4] and SuSiE [1] run on East Asians and Europeans, individually (Fig E). The SuSiE method is a new approach to fine mapping that has a different model than the other methods, so we first discuss the rest of the methods and then return to SuSiE.

All other methods were run with posterior probability threshold $\rho^* = 0.95$, so methods with 95% or higher sensitivity were considered "well-calibrated" (dashed line in the bar plots). MsCAVIAR's heterogeneity parameter was set to $\tau^2 = 0.5$ (Methods); the sensitivity of MsCAVIAR's performance to different settings of this parameter is shown in the appendix (Fig G). All methods but SuSiE

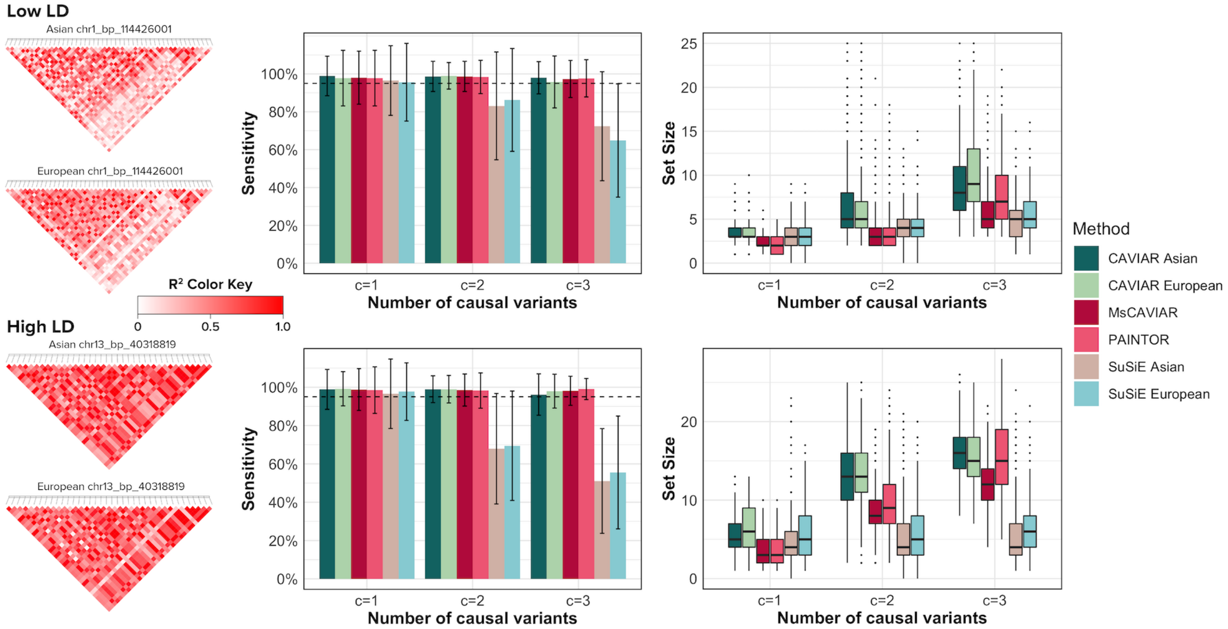

Fig E. Comparison of sensitivity and set size using simulated data. Comparison of the methods with c=1, c=2, or c=3 causal variants implanted. Both low LD (top half) and high LD (bottom half) settings were evaluated. The bar plots (left) display the sensitivity of the methods, with standard deviation bars included. The dashed line reflects the expected posterior probability of recovering all causal SNPs; methods that reach this threshold are considered "well-calibrated". The box plots (right) show the set sizes returned by the methods; for SuSiE, this is calculated as the sum of the sizes of credible sets returned. The boxes represent the interquartile range of causal set sizes identified by each tool, the lines inside the boxes represent the median, and the whiskers extend to the non-outlier extremes. Outliers are represented as dots above or below the whiskers. SuSiE's credible sets differ from the causal sets of the other methods in that SuSiE does not attempt to capture all causal SNPs, so the sensitivity calibration is not directly comparable to the other methods.

were well-calibrated in both LD settings (Fig E, bar plots). This is unsurprising since CAVIAR is well-known to be calibrated in the single study setting [4, 5, 6], as is PAINTOR in the trans-ethnic setting [3].

However, when considering the subset of simulations in which each method was able to correctly capture all causal variants (i.e. 100% sensitivity), we observed that MsCAVIAR consistently returns the smallest average set size (Fig E, box plots). MsCAVIAR and PAINTOR return smaller set sizes than CAVIAR run on either population across all settings, highlighting the value of using varying LD patterns in different populations to refine fine-mapping results. MsCAVIAR returned smaller set sizes than PAINTOR with multiple causal variants or high LD. This may be due to MsCAVIAR's explicit modeling of heterogeneity between studies. In both the high LD and multiple causal variants setting, complex and strong correlations between non-causal and causal SNPs are induced, and modeling heterogeneity between studies allows for more effective use of the differing LD structures to disentangle non-causal from causal SNPs.

SuSiE [1] takes a different approach to fine mapping from the other methods. Instead of returning a causal set, SuSiE returns (potentially multiple) credible sets for a locus, each of which is

expected to contain at least one causal SNP. The goal of SuSiE is not to capture all causal variants in a locus, but to return one or more minimal size credible sets, each of which has ρ probability of containing at least one true causal effect. This explains why SuSiE is not well-calibrated according to our causal set definition, which expects all casual variants to be captured with probability ρ^*, when there is more than one causal SNP. It is worth noting, however, that SuSiE's credible set is equivalent to the causal set (as defined by the other methods) when the methods assume that there is only one causal SNP in a locus. In this case, all methods are well-calibrated and MsCAVIAR returns the smallest average set size, followed by PAINTOR, then SuSiE, and finally CAVIAR. This finding supports the hypothesis that using trans-ethnic information improves fine mapping resolution.

3 MsCAVIAR is well-calibrated with different population sizes between studies

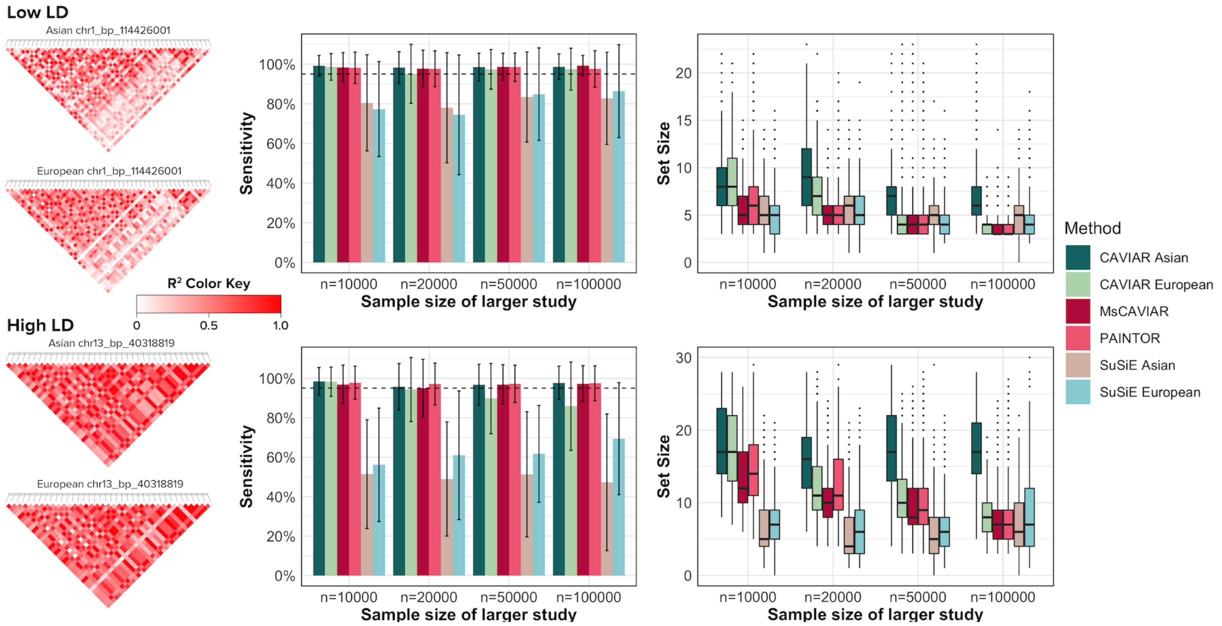

Fig F. Comparison of sensitivity and set size using simulated studies with unequal sample sizes. Comparison of the methods with 3 causal variants implanted and imbalanced sample sizes. The size of the Asian population was fixed at 10,000, while the European study was set to be 1, 2, 5, or 10 times larger. Both low LD (top half) and high LD (bottom half) settings were evaluated. The bar plots (left) display the sensitivity of the methods, with standard deviation bars included. The dashed line reflects the expected posterior probability of recovering all causal SNPs; methods that reach this threshold are considered "well-calibrated". The box plots (right) show the set sizes returned by the methods; for SuSiE, this is calculated as the sum of the sizes of credible sets returned. The boxes represent the interquartile range of causal set sizes identified by each tool, the lines inside the boxes represent the median, and the whiskers extend to the non-outlier extremes. Outliers are represented as dots above or below the whiskers. SuSiE's credible sets differ from the causal sets of the other methods in that SuSiE does not attempt to capture all causal SNPs, so the sensitivity calibration is not directly comparable to the other methods.

It is possible that input studies can have different sample sizes, in which case the non-centrality parameters of their SNPs are expected to be different proportionally to sample size, in addition to heterogeneity. We tested whether MsCAVIAR would still be well-calibrated in this setting, and compared it again with trans-ethnic PAINTOR and with CAVIAR and SuSiE run on the individual populations (Fig F). The caveats with SuSiE discussed in the previous section also apply here, so we omit it from further discussion and refer readers to the appendix.

In order to evaluate performance under this scenario in a simulation study, we used the same LD matrices from the previous section, but now varied the population size for one of the studies. We fixed the population size of the Asian study at 10,000 individuals, and varied the European study to have population sizes of 1, 2, 5, or 10 times that of the Asian study. Consequently, the effect sizes of causal SNPs in the European study were larger than those of the corresponding SNPs in the Asian study by a factor of $\sqrt{1}$, $\sqrt{2}$, $\sqrt{5}$, and $\sqrt{10}$ (Methods). For the sake of sufficient statistical power, we ensured that the causal variants in the smaller study were still statistically significant genome-wide. 1000 simulation replicates were run for each LD setting. In each simulation, we implanted three causal SNPs and simulated their effect sizes, with the association statistics of non-causal SNPs being based on their correlation with causal SNPs (Methods). All methods were run with posterior probability threshold $\rho^* = 0.95$, so methods with 95% or higher sensitivity were considered "well-calibrated" (dashed line in the bar plots). MsCAVIAR was run with its heterogeneity parameter set at $\tau^2 = 0.5$ (Methods).

Once again, MsCAVIAR was well-calibrated and generally returned the smallest causal set sizes. As the sample size differences grew, the difference between MsCAVIAR, CAVIAR on Europeans, and PAINTOR tended to diminish. This is likely due to the fact that we required SNPs to be genome-wide significant in the smaller study, such that the larger study had very large effect sizes for causal SNPs when there was a significant sample size imbalance, making the fine mapping problem easier. Reinforcing this interpretation is the fact that CAVIAR on Asians had consistently much larger causal set sizes than the other methods when the sample size imbalance was large.

All methods (exempting SuSiE) were well-calibrated in the low LD setting, but we observed that as the sample size increases with high LD that CAVIAR's calibration on the larger population decreases. This is likely due to the extremity of the situation, with exceptionally large effect sizes in combination with the high LD setting. We again, note that SuSiE's miscalibration is due to fundamental differences between SuSiE and the other methods.

4 Effects of heterogeneity parameter on MsCAVIAR

To examine the effects of mismatched true and model heterogeneity on MsCaviar results, we first simulated our studies with different "true heterogeneity" τ^2. We used two regions from the 1000 Genomes project [2] to generate a high LD matrix and low LD matrix. For the low LD matrix, 20% of the SNPs have LD equal to or higher than 0.5, while the region with relatively high LD have 80% of the SNPs with LD equal to or higher than 0.5. We pruned groups of SNPs that were in perfect LD in one or more of the populations, leaving one SNP for each. The low LD matrix contains 48 SNPs, and the high LD matrix contained 38 SNPs Using these two matrices LD matrices, we implanted causal SNPs and simulated their effect sizes. In each simulation, we implanted either 1, 2, or 3 causal SNPs.

Each casual SNP's true overall non-centrality parameter Λ was drawn according to $\mathcal{N}(5.2, 0.125^2)$. The study specific non-centrality parameter Λ_i for each study i was drawn according to $\Lambda_i \sim \mathcal{N}(\Lambda, \tau^2)$, where $\tau^2 = 0.5$, 1, or 2. For each model configuration, we performed 1000 replicate simulations (e.g. re-drawing the causal SNP effect sizes and re-picking the causal SNPs). We then

ran MsCAVIAR with different modeled heterogeneity settings, $\tau^2 = 0.5$, 1, or 2, on the simulations with the various true heterogeneity settings (Fig G). MsCAVIAR was well-calibrated and maintained similar set sizes even when the modeled heterogeneity did not match the true heterogeneity, indicating that MsCAVIAR is fairly robust to small mis-specifications of the τ^2 parameter.

5 Out-of-sample LD matrices degrade the accuracy of fine-mapping

The methods CAVIAR [4], PAINTOR [3], and MsCAVIAR are designed such that they only require the z-scores as summary data and may use an external LD matrix representative of the samples for fine-mapping. Previous work has shown that this approach is underpowered [7]. In the results section "MsCAVIAR improves fine mapping resolution in a simulation study" we provide each fine-mapping method the LD matrices generated in-sample. Here, we simulate data in an almost identical fashion, but we generate the input LD matrices using the 1000 Genomes Project [2] to demonstrate the impact of the out-of-sample LD matrices on our fine-mapping results. We exclude SuSiE [1] from this set of analyses as the method was not designed to accept summary data.

For the results shown in Fig H, we use two sets of 9,000 unrelated individuals from the UK Biobank with European and Asian ancestry and then identify the corresponding populations in 1000 Genomes Project to generate the out-of-sample LD matrices. For the 9,000 UK Biobank samples with European ancestry, we select the 503 samples in the super population "EUR" in the 1000 Genomes Project as the reference sample. For the individuals with Asian ancestry, we needed to use two super populations "SAS" and "EAS" due to the UK Biobank sample containing 1600 individuals with Chinese ancestry, 5900 individuals with Indian ancestry, and 1800 individuals with other Asian ancestry. We generate our representative sample using all 489 "SAS" individuals and 123 "EAS" individuals sampled across subpopulations. This sub-sampling was done to approximate the proportion of individuals with Chinese and Indian ancestry. We note that while our example of out-of-sample LD for Asian ancestry is more extreme than when only 1 "super population" is used, it highlights how the accuracy of the out-of-sample LD impacts fine-mapping.

We looked at the three loci with low, medium, and high LD described in "MsCAVIAR improves fine mapping resolution in a simulation study" and retained the SNPs with MAF > 0.05 in the two samples generated from the 1000 Genomes Project. This resulted in 144, 125, and 149 SNPs for the low, medium, and high LD loci, respectively. Using this set of SNPs, we simulate the causal variants identically to the process described in the main results section referenced above. The only difference was that we provide the LD matrices generated from the 1000 Genomes samples to the methods instead of their in-sample LD matrices.

In the results shown in Fig H, we compare MsCAVIAR to trans-ethnic PAINTOR [3] and to CAVIAR [4] run on the Asian and European populations, separately. We average the results over the set of simulations for each number of causal SNPs: 1, 2, and 3. All methods were run using $\rho^* = 0.95$ as the posterior probability threshold; therefore methods were considered "well-calibrated" if their sensitivity was at least 95% (dashed line in Fig HA), and we set MsCAVIAR's heterogeneity parameter to $\tau^2 = 0.52$ (see Methods). We evaluated the precision of the methods in Fig HB where we show the causal set size. This metric is informative when the method returns the causal variant(s) because then fewer non-causal variants or "false positives" are being returned in the set.

When there is only 1 causal SNP, all methods are well-calibrated; however, only CAVIAR when analyzing European samples is well-calibrated when there are 3 causal SNPs and is slightly below the threshold for 2 implanted causal variants (94.3%). All other methods see a serious degradation in their sensitivity. This decrease in performance is a result of a poor approximation to the LD

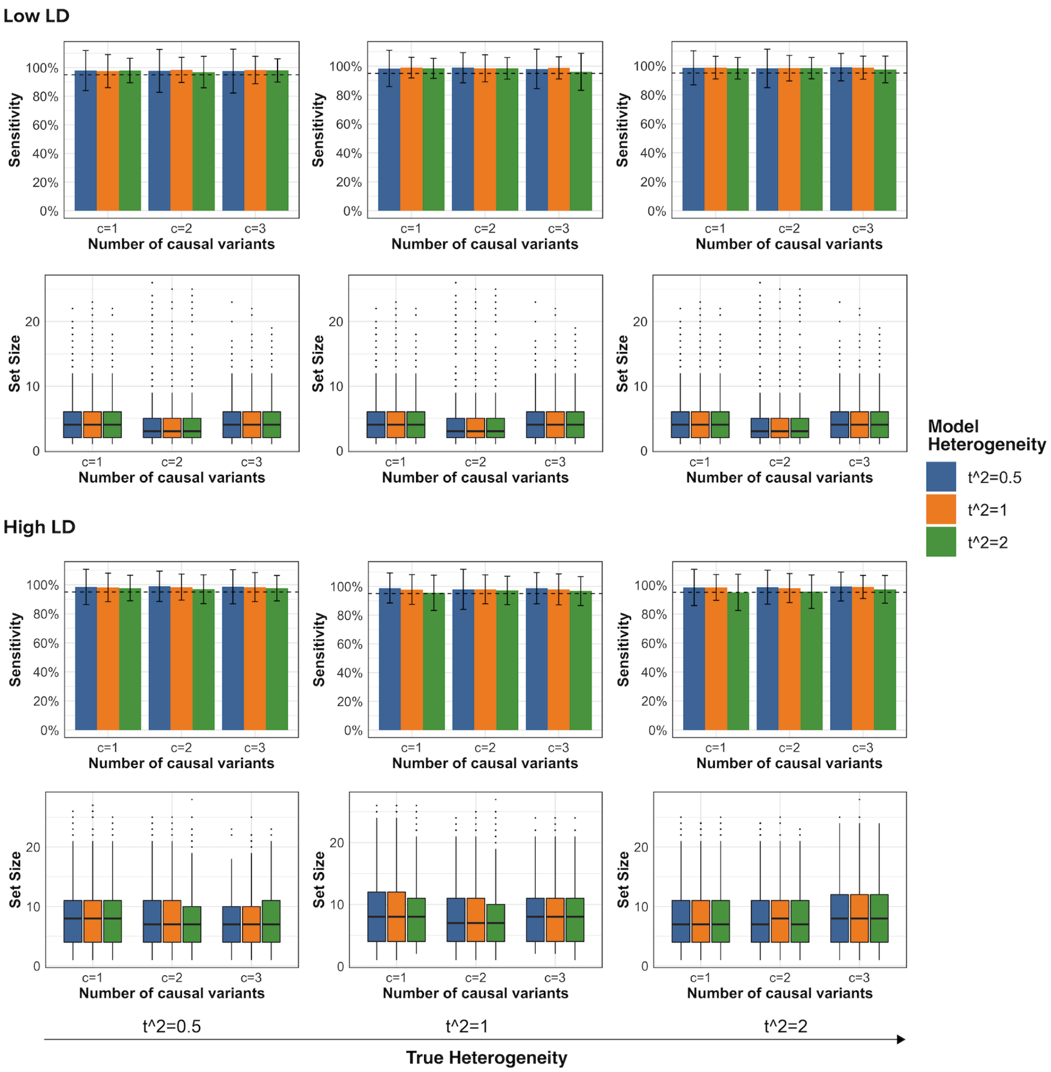

Fig G. Evaluation of the sensitivity and set sizes of MsCAVIAR results under mis-specified heterogeneity parameters. Each column of plots shows a different "True Heterogeneity" value τ^2 used to simulate Z-scores of causal variants. Different-colored bars/boxes correspond to different values of τ^2 used internally in MsCAVIAR's model, referred to as the "Model Heterogeneity". The model is mis-specified when the Model Heterogeneity does not match the True Heterogeneity. The first two rows of plots are based on a low LD locus, and the bottom two rows are based on a high LD locus. The bar plots (1st and 3rd rows) display the sensitivity of the results, with standard deviation bars included. The dashed line reflects the expected posterior probability of recovering all causal SNPs; methods that reach this threshold are considered "well-calibrated". The box plots (2nd and 4th rows) show the set sizes returned by MsCAVIAR. The boxes represent the interquartile range of causal set sizes identified by each tool, the lines inside the boxes represent the median, and the whiskers extend to the non-outlier extremes. Outliers are represented as dots above or below the whiskers. Simulations were performed with c=1, c=2, or c=3 causal variants.

matrix for individuals with Asian ancestry. When the out-of-sample LD accurately reflects the sample, as is the case for European ancestry, CAVIAR returns results comparable to when an in-

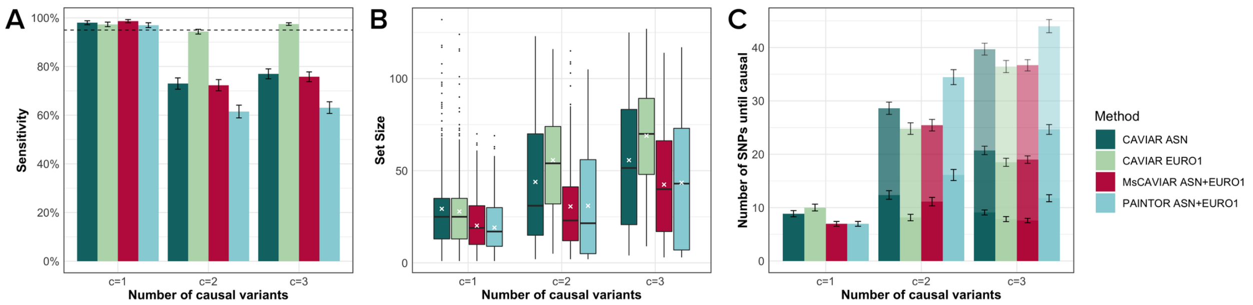

Fig H. Comparison of sensitivity, precision, and set sizes using simulated data and out-of-sample LD matrices. We compare MsCAVIAR, PAINTOR, and CAVIAR with $c \in \{1, 2, 3\}$ causal variants averaging over 3 loci and 5 levels of heritability with 20 replicates for each value of c. (A) Bar graph indicating the sensitivity of the method and the expected posterior probability, ρ, of recovering all causal SNPs represented as a dashed line. (B) Box plots showing the average set sizes each method returns. Each box is the interquartile range of causal set sizes. The middle black line represents the median and the white crosses indicating the mean. (C) Bar graph displaying the average number of SNPs in descending order of posterior inclusion probability (PIP) until 1,2, or 3 causal SNPs is identified. Stacked bars represent increasing numbers of causal SNPs identified until the true number of causal SNPs (x-axis) are identified.

sample LD matrix is provided. For MsCAVIAR and PAINTOR, however, we see incorporating two populations does not help when one sample's LD is poorly approximated. Though the set sizes are smaller in Fig H, the specificity is also lower than either run of CAVIAR. While MsCAVIAR and PAINTOR are both poorly calibrated, we see that MsCAVIAR is more robust to the out-of-sample LD than PAINTOR. Further work would need to be done to explore this phenomenon.

At present, we encourage users to use the in-sample LD matrix whenever possible. If this is not a possibility, we advise the user to interpret their results with the understanding the out-of-sample LD may fail to provide well-calibrated results, and the quality of results depend how well the out-of-sample LD approximates the in-sample LD. Future work could also enable the method to incorporate the sufficient summary data described in [7].

6 Loci and set sizes for the real data fine-mapping analysis.

Below we include a table of the real data results. The first column is the list of regions fine-mapped. The remaining four columns is the number of SNPs returned by each method's causal set. The methods in order are: CAVIAR on Biobank Japan (BBJ) "CAVIAR-Asian", CAVIAR on UK Biobank (UKB) "CAVIAR-Euro", PAINTOR, and MsCAVIAR. The last two methods fine-map utilizing data from both BBJ and UKB.

**LOCUS**	**CAVIAR-Asian**	**CAVIAR-Euro**	**PAINTOR**	**MsCAVIAR**
chr10:113440329-114440329	83	52	58	61
chr10:115286236-116286236	31	16	18	10
chr10:122398697-123398697	97	85	53	102
chr10:33136099-34136099	230	32	48	20
chr10:45522005-46522005	59	48	38	34
chr10:64552205-65552205	275	249	230	238

LOCUS	CAVIAR-Asian	CAVIAR-Euro	PAINTOR	MsCAVIAR
chr10:88011326-89011326	88	77	54	63
chr10:94314710-95314710	148	15	22	6
chr10:94816037-95816037	112	7	17	4
chr10:99269388-100269388	66	57	57	55
chr11:109470749-110470749	72	65	62	62
chr11:115908029-116908029	9	6	6	5
chr11:117920241-118920241	54	46	44	38
chr11:122014403-123014403	97	59	58	43
chr11:125725876-126725876	143	50	47	18
chr11:13837575-14837575	254	151	118	93
chr11:14365399-15365399	137	112	105	71
chr11:27248493-28248493	121	101	90	90
chr11:45413607-46413607	15	13	15	12
chr11:46228966-47228966	170	57	16	24
chr11:46837383-47837383	396	82	81	73
chr11:47398535-48398535	248	34	49	36
chr11:47944711-48944711	71	9	4	9
chr11:61092362-62092362	44	6	25	6
chr11:63504723-64504723	23	14	18	16
chr11:64973798-65973798	100	62	65	64
chr11:65566993-66566993	35	27	33	28
chr11:68116074-69116074	55	51	50	46
chr11:74952486-75952486	143	116	110	100
chr12:109371179-110371179	232	177	19	28
chr12:110558188-111558188	64	88	35	65
chr12:111218231-112218231	10	172	5	7
chr12:112091686-113091686	16	27	27	19
chr12:112819105-113819105	6	90	10	4
chr12:120916622-121916622	159	110	94	96
chr12:122688475-123688475	129	31	19	19
chr12:123236084-124236084	430	253	128	249
chr12:123909502-124909502	98	56	6	17
chr12:124765201-125765201	19	6	4	5
chr12:19970199-20970199	75	9	19	9
chr12:53233529-54233529	33	23	25	17
chr12:56831741-57831741	42	19	29	28
chr12:57339173-58339173	46	20	16	15
chr12:57922642-58922642	36	33	34	33
chr12:6163964-7163964	89	81	79	69
chr12:70973887-71973887	17	14	16	14
chr12:8595905-9595905	83	51	42	54
chr14:102747844-103747844	22	17	16	10
chr14:104758892-105758892	33	8	26	8
chr14:68649372-69649372	29	28	30	26
chr14:73750126-74750126	30	22	25	19
chr14:74860906-75860906	367	320	236	283
chr14:81135888-82135888	50	49	47	39

LOCUS	CAVIAR-Asian	CAVIAR-Euro	PAINTOR	MsCAVIAR
chr15:23444489-24444489	25	21	21	21
chr15:42599550-43599550	99	83	55	45
chr15:57703414-58703414	8	6	5	3
chr15:58223939-59223939	5	3	6	3
chr15:59004897-60004897	30	52	20	14
chr15:62844167-63844167	18	12	16	12
chr15:74212937-75212937	219	191	171	189
chr15:74964992-75964992	51	42	42	40
chr16:53303187-54303187	59	45	47	40
chr16:55410194-56410194	51	8	6	5
chr16:55918284-56918284	50	10	7	7
chr16:56995026-57995026	4	3	5	6
chr16:66898369-67898369	57	38	48	43
chr16:67472194-68472194	301	154	8	94
chr16:68059767-69059767	302	111	5	82
chr16:74682354-75682354	311	295	254	286
chr16:81034790-82034790	86	17	21	19
chr17:37310218-38310218	287	111	42	110
chr17:37844485-38844485	192	94	58	91
chr17:40281561-41281561	196	182	64	66
chr17:41426126-42426126	22	7	7	6
chr17:66323805-67323805	129	36	27	19
chr17:6962969-7962969	34	30	28	22
chr17:75898130-76898130	157	112	124	97
chr17:7590908-8590908	32	25	27	23
chr18:19166877-20166877	147	137	140	138
chr18:46078242-47078242	49	5	12	4
chr18:46609955-47609955	7	13	7	6
chr18:47132322-48132322	24	15	6	7
chr18:57413965-58413965	65	41	48	33
chr19:10850488-11850488	28	14	19	8
chr19:33396432-34396432	65	30	40	27
chr19:44911941-45911941	6	3	5	3
chr19:45884830-46884830	72	45	39	41
chr19:51841757-52841757	291	204	100	149
chr19:54300500-55300500	14	14	6	11
chr19:7011798-8011798	16	13	13	11
chr19:7929323-8929323	46	6	7	5
chr1:109166352-110166352	41	16	25	15
chr1:109732983-110732983	85	12	7	7
chr1:178013895-179013895	193	138	127	123
chr1:181671957-182671957	77	67	70	63
chr1:219182178-220182178	24	19	19	15
chr1:220470028-221470028	51	24	30	19
chr1:229797778-230797778	177	19	11	23
chr1:230323363-231323363	53	6	22	6
chr1:23210475-24210475	26	19	21	18

LOCUS	CAVIAR-Asian	CAVIAR-Euro	PAINTOR	MsCAVIAR
chr1:39503410-40503410	183	103	84	115
chr1:435222-1435222	13	8	11	8
chr1:65573952-66573952	23	14	18	12
chr2:-216206-783794	73	64	52	55
chr1:93358292-94358292	274	156	48	56
chr20:17096155-18096155	70	53	50	31
chr20:44051855-45051855	75	10	4	12
chr20:45840596-46840596	138	7	12	7
chr20:50746378-51746378	48	44	42	41
chr20:61847191-62847191	255	183	9	87
chr21:45771452-46771452	122	127	71	61
chr21:46399279-47399279	60	52	51	47
chr22:21425017-22425017	69	68	68	68
chr22:29924863-30924863	260	206	187	188
chr22:30425616-31425616	217	170	128	159
chr22:38099857-39099857	31	23	24	16
chr22:38610124-39610124	33	24	29	23
chr2:100295962-101295962	60	59	4	49
chr2:134763081-135763081	96	85	81	76
chr2:135407088-136407088	60	59	57	53
chr2:135907479-136907479	26	24	24	22
chr2:136507429-137507429	21	19	20	18
chr2:165055539-166055539	144	73	55	61
chr2:173416114-174416114	78	57	67	50
chr2:19871772-20871772	13	10	12	10
chr2:20731524-21731524	105	24	32	27
chr2:211040507-212040507	64	55	46	40
chr2:226599180-227599180	218	61	107	139
chr2:241737902-242737902	105	87	71	75
chr2:48462291-49462291	31	26	29	23
chr3:11851223-12851223	365	79	32	69
chr3:135380410-136380410	130	100	91	103
chr3:135904095-136904095	163	130	115	123
chr3:156298732-157298732	46	23	35	24
chr3:184782232-185782232	66	55	64	60
chr3:185322353-186322353	92	10	29	10
chr3:46425539-47425539	152	126	110	112
chr3:47221512-48221512	67	66	57	55
chr3:49524027-50524027	249	163	164	100
chr4:102181041-103181041	23	14	19	12
chr4:102688709-103688709	24	10	16	12
chr4:25550450-26550450	11	3	7	3
chr4:39288949-40288949	91	77	68	59
chr4:470112-1470112	63	56	50	44
chr4:54998781-55998781	25	20	23	21
chr4:68861445-69861445	29	25	27	24
chr4:86973776-87973776	133	101	100	84

LOCUS	CAVIAR-Asian	CAVIAR-Euro	PAINTOR	MsCAVIAR
chr4:87518991-88518991	242	68	103	89
chr4:89230074-90230074	111	65	78	65
chr4:99761577-100761577	160	110	100	83
chr5:103434169-104434169	44	35	26	23
chr5:110726812-111726812	79	73	69	63
chr5:126850549-127850549	49	35	21	19
chr5:157499022-158499022	122	109	71	87
chr5:55304552-56304552	88	9	6	9
chr5:74503678-75503678	78	58	49	41
chr7:551664-1551664	195	85	65	51
chr7:106289152-107289152	63	41	46	34
chr7:129938531-130938531	70	31	37	29
chr7:150040196-151040196	407	203	105	187
chr7:17420253-18420253	359	230	221	252
chr7:25897239-26897239	73	54	53	48
chr7:35710924-36710924	114	80	86	75
chr7:49806810-50806810	32	32	34	30
chr7:5970622-6970622	111	82	54	66
chr7:72539406-73539406	117	68	90	78
chr7:79842775-80842775	148	173	68	69
chr8:103383630-104383630	45	2	35	11
chr8:116103103-117103103	216	113	24	110
chr8:12123463-13123463	24	21	24	22
chr8:8683358-9683358	96	10	24	9
chr8:121367780-122367780	79	70	15	25
chr8:125980367-126980367	73	36	40	27
chr8:143806597-144806597	58	35	40	33
chr8:18751679-19751679	13	7	5	4
chr8:19324667-20324667	55	24	6	18
chr8:19941920-20941920	10	3	6	3
chr8:70838185-71838185	325	314	298	285
chr8:9277873-10277873	51	12	24	9
chr9:106574213-107574213	8	22	9	14
chr9:107161742-108161742	6	3	5	3
chr9:123131225-124131225	109	121	70	103
chr9:139487756-140487756	38	32	33	31
chr9:14804782-15804782	52	11	14	10
chr9:94882297-95882297	121	101	98	96

Table A. Loci and causal set sizes for the trans-biobank High Density Lipoprotein fine mapping analysis. Each row is a fine mapping locus, and the columns represent the number of SNPs in the causal sets returned by each method.

References

1. Wang G, Sarkar A, Carbonetto P, Stephens M. A simple new approach to variable selection in regression, with application to genetic fine mapping. Journal of the Royal Statistical Society: Series B (Statistical Methodology). 2020;82(5):1273–1300. doi:https://doi.org/10.1111/rssb.12388.

2. Consortium GP, et al. A global reference for human genetic variation. Nature. 2015;526(7571):68.

3. Kichaev G, Pasaniuc B. Leveraging functional-annotation data in trans-ethnic fine-mapping studies. The American Journal of Human Genetics. 2015;97(2):260–271.

4. Hormozdiari F, Kostem E, Kang EY, Pasaniuc B, Eskin E. Identifying causal variants at loci with multiple signals of association. Genetics. 2014; p. genetics–114.

5. Chen W, Larrabee BR, Ovsyannikova IG, Kennedy RB, Haralambieva IH, Poland GA, et al. Fine mapping causal variants with an approximate Bayesian method using marginal test statistics. Genetics. 2015;200(3):719–736.

6. Benner C, Spencer CC, Havulinna AS, Salomaa V, Ripatti S, Pirinen M. FINEMAP: efficient variable selection using summary data from genome-wide association studies. Bioinformatics. 2016;32(10):1493–1501.

7. Lee Y, Luca F, Pique-Regi R, Wen X. Bayesian Multi-SNP Genetic Association Analysis: Control of FDR and Use of Summary Statistics. Biorxiv. 2018;doi:10.1101/316471.
