# Supplementary material for: Identifying causal variants by fine mapping across multiple studies
Source: PLoS Genet. 2021 Sep 20;17(9):e1009733. doi: 10.1371/journal.pgen.1009733 (PMC8491908; doi:10.1371/journal.pgen.1009733)
Supplement: Attachment — Submitted filename: mscaviar_response_to_reviewers.pdf [file pgen.1009733.s002.pdf]

UNIVERSITY OF CALIFORNIA, LOS ANGELES

BERKLEY • DAVIS • IRVINE • LOS ANGELES • MERCED • SANTA BARBARA
RIVERSIDE • SAN DIEGO • SAN FRANCISCO • SANTA CRUZ

May 17th, 2021

Dear Dr. Zeggini and Dr. Balding,

Thank you for considering our manuscript, “Identifying Causal Variants by Fine Mapping Across Multiple Studies,” for publication in PLoS Genetics. We would like to thank the reviewers for their constructive feedback. Following the reviewers’ suggestions, we have made extensive edits to our manuscript, primarily to the results, methods, and discussion sections. Details are included below with inline responses to the reviewers, with new text in red. However, as the details are quite extensive, we thought it would be helpful to provide a summary here.

The Results section is almost entirely new. Following the reviewers’ suggestions, we changed our simulation approach to use genotypes from the UK Biobank. We generate summary statistics by simulating phenotypes with GCTA based on our simulated causal SNP effect sizes, followed by using fastGWA to generate the GWAS summary statistics. We believe that this addresses several comments by the reviewers regarding the realism of the summary statistics and locus sizes from the previous simulations. The real data analysis was modified to use 1 Mb loci instead of 100 Kb loci, and the filtering criteria were relaxed. Because this resulted in very few available loci for the Type 2 Diabetes analysis, we removed that analysis and instead focused on the High Density Lipoprotein analysis. In both the simulated and real data, we removed the LD pruning step, following the reviewers’ suggestions.

Using unpruned loci caused issues with inverting low rank LD matrices, so we implemented a computational method to allow for computing the Multivariate Normal likelihood function even when the LD matrix is low rank. To our knowledge, this method has been suggested previously, but never implemented in fine mapping software. The Methods section was amended to include this extension. Additionally, following comments from the reviewers, we added a new overview subsection to the methods section that we hope clarifies the method. We also made substantial edits to several other Methods subsections in response to reviewer comments.

Finally, the reviewers made a number of excellent suggestions regarding better usage of MsCAVIAR when there are multiple studies from the same ethnicity, as well as several possible extensions to the method. While we were unable to implement all of these extensions, we considerably expanded the Discussion section to address all of the salient issues, in hopes that they can inspire future work.

We would like to thank the reviewers for their comments, as we believe that the paper and method are stronger as a result of addressing them. We would also like to thank the editors again for considering our revised manuscript.

Sincerely,

Eleazar Eskin

Professor

University of California, Los Angeles

Response to Reviewers' Comments

Reviewer 1 Remarks

> *Reviewer #1: Summary: Minority population GWAS and trans-ethnic finemapping have increased in popularity due to the diversity in LD patterns across populations and how this diversity can be exploited to improve fine-mapping resolution. To this end, the authors present the statistical finemapping method "MsCAVIAR", which is an extension of the previously developed Bayesian fine-mapping software CAVIAR. The primary advantage and novelty of the proposed approach is that it simultaneously addresses both effect heterogeneity and multiple causal variants at a given locus of interest for trans-ethnic finemapping. The authors' perform a number of simulation studies as well as real data application to demonstrate the performance of their approach against leading methods that accommodate multiple causal variants. The method performs as well or better than its primary competitor (PAINTOR) in many regards. Overall, the paper is clear and well-written. The authors' carefully outline their statistical methodology, addressing a number of computational hurdles. They address a number of limitations in their assumptions and highlight the strengths/weaknesses of their method. The authors also make code publicly available on Github, which appears to be complete and well-documented. I do have some questions regarding computational efficiency, and there are some issues with respect to consistency of notation – although most of my comments are minor.*

Reviewer 1, Specific Comment #1

> *As the authors' note, there are a few limitations on their assumptions regarding tau and how it is fixed a priori in application. This corresponds both across loci and across studies, where imbalance in contributing studies vis a vis ancestral population may be a concern (as published GWAS are in predominantly European populations). Regarding the latter, one advantage of MR-MEGA in trans-ethnic finemapping is how it leverages meta-regression to account for distribution of genetic ancestry across the contributing individual studies, thus decomposing the effect heterogeneity into ancestral and random components. To this end, the authors suggest selecting one study from each population for inclusion. However, this naturally reduces finemapping efficiency depending on the distribution of study sizes. Would an alternative strategy using MsCAVIAR be to initially conduct fixed-effects meta-analyses within homogenous populations, where warranted, and then combine diverse population results for trans-ethnic finemapping?*

Authors' Response

We thank the reviewer for raising this issue to our attention. We have changed the text to reflect this recommendation. The text now reads (see discussion, third paragraph):

We also assume that all studies are drawn with equal heterogeneity τ^2. This is unlikely to be true if multiple studies are from a single population while another study is from a different population. In such a scenario, we recommend grouping the studies by population, running fixed effects meta-analysis on each group, and then running MsCAVIAR on the results for the different groups. Concretely, the input summary statistics for MsCAVIAR should be the results from the meta-analysis of each population, and the input LD matrices should be derived from either the genotype data (if available) or the appropriate reference panels for each population.

Reviewer 1, Specific Comment #2

Speaking of which – the authors initially mention MR-MEGA in the introduction but do not discuss it any further or include it in their comparisons. Given that the MR-MEGA paper demonstrates improved finemapping over PAINTOR in their paper, I assume the rationale for its exclusion for comparative performance analyses is due to its limiting assumption regarding number of underlying causal variants?

Authors' Response

We thank the reviewer for raising this issue. The limitation to modeling a single causal SNP is indeed a major reason why MR-MEGA was not included. Additionally, MR-MEGA requires different input files, gives different output statistics, and employs a substantially different generative model for the observed data than MsCAVIAR. Overall, we do not believe that it would be an apples-to-apples comparison.

Reviewer 1, Specific Comment #3

How would the authors additionally integrate functional annotation into their fine-mapping method, similar to PAINTOR?

Authors' Response

We thank the reviewer for raising this issue. MsCAVIAR can, in principle, incorporate functional priors by allowing the prior probabilities of the SNPs being causal to be different, as discussed in CAVIAR; currently, as you can see, they are all set to a fixed "gamma" prior probability. However, we are concerned that setting these priors arbitrarily is dangerous, and that more work would be needed to determine how best to model various functional priors in the context of MsCAVIAR's model. We think that this could potentially be a good direction for future research. Thus, we have included the following in our discussion:

Functional information can in principle be factored into MsCAVIAR's model by modifying the prior distribution P(C) so that not every variant has the same prior probability of being causal, as described in the CAVIAR paper \cite{caviar}. However, setting these priors arbitrarily can yield

misleading results, and future work is needed to determine how best to model various functional priors in the context of MsCAVIAR's model.

Reviewer 1, Specific Comment #4

> *It may be useful to quickly mention in "Parameter setting in practice" the relevance of 5.2 as a Z-score in relation to the traditional genome-wide significance criterion (i.e., the corresponding p under two-sided Wald test would be ~5-e08).*

Authors' Response

Thank you for drawing our attention to this. We have added the following line in the "Parameter setting in practice" section:

> This value corresponds to the traditional genome-wide significant Z-score of 5.2, for which the two-sided Wald test p-value is 5×10^{-8}, which is considered significant by (conservatively) correcting for multiple testing [35].

Reviewer 1, Specific Comment #5

> *Similarly - I'm not finding any clear justification for MsCAVIAR's default value of tau2 = 0.52 – I assume the connection lies with just taking forcing a mean/variance relationship in effect heterogeneity for Z-scores at the genome-wide significance threshold?*

Authors' Response

We thank the reviewer for raising this concern. The specific value is somewhat arbitrary -- it is 10% of the default sigma value of 5.2 -- but it was chosen to be large enough to detect heterogeneity but not so large as to make small amounts of heterogeneity difficult to detect. We also show in Fig. S7 that MsCAVIAR is relatively robust to small misspecifications in the tau^2 parameter. We have modified the "Parameter Setting in Practice" subsection as follows, with new text in red and existing text (included for context) in black:

> This value of σ^2_g may not represent the actual heritability partitioning, but we set the parameter this way in our method for the practical purpose of giving MsCAVIAR power to fine map borderline significant variants in the smallest study. Similarly, we set $\tau^2=0.52$ by default, e.g. 10% of the value of $\sigma^2_g n_m$, with the value chosen to give power to detect both small and large amounts of heterogeneity. We empirically observed that small misspecifications in the heterogeneity parameter do not substantially adversely affect results (Fig. S7).

Reviewer 1, Specific Comment #6

Equation 10. It's kind of confusing to use T to denote both the number of studies as well as the transpose operator. Similarly, later in the methods n is used to denote the number of studies, but also the number of subjects within a study. And then again it seems that 'm' is used to index study in "Extending MsCAVIAR to different sample sizes". Some care should be used in maintaining consistent notation.

Authors' Response

We thank the reviewer for pointing out these discrepancies. In order to standardize all of the notation while minimizing the number of different letters used, we have changed the notation in all sections to the following:

- "M" is the number of SNPs per study, and we use "m" to refer to specific SNPs
- "N" is the number of people per study (sample size), and we use "n" to refer to specific individuals
- "Q" is the number of studies, and we use "q" to refer to a specific study
- "K" is the number of causal SNPs per study

Reviewer 1, Specific Comment #7

*It's not immediately clear how the computational cost scales with respect to number of included studies. Given the single study setting is O(k^3) plus some O(mk^2) – do we replace k with (k*n) to get the computational costs, where n = # of studies? Thus, is # of studies a similarly strong limiting factor in computational burden as k, which would potentially motivate the population-wise study aggregation study mentioned above?*

Authors' Response

We thank the reviewer for raising this question. The computational complexity does indeed depend on the number of studies. This is mentioned at the end of the "Efficient meta-analysis" sub-section (using the notation mentioned above, where Q is the number of studies, M is the number of SNPs, and K is the number of causal SNPs):

> The computation time is thus reduced from O(M^3 * Q^3) to O(K^3 * Q^3).

The reviewer is referring to the notation in the single-study setting, in the "Efficient computation of likelihood functions" subsection. We surmised that it would be helpful if we explicitly linked the two sections together, so we have added the following sentence to the end of this subsection:

> In the "Efficient meta-analysis" subsection below, we discuss the computational complexity and the use of these efficient matrix computations in the multiple study setting.

We emphasize that the above notation refers to the computational complexity of specific matrix operations that occur during the likelihood function computation for a specific causal configuration, not the method's overall computational complexity. The method's overall computational complexity can't be captured easily in a single big-O expression because it also depends a great deal on the locus itself -- how many SNPs there are, the LD structure, and how many causal SNPs there *actually* are -- as well as the parameters supplied by the user, chiefly the maximum number of causal SNPs allowed and the posterior probability threshold for termination. The chief determinant of the runtime is the number of SNPs in the locus and (especially) the maximum number of causal SNPs allowed, because, if there are M SNPs and up to K may be causal, there are potentially up to "M choose K" causal status vectors to evaluate. The number of studies can potentially be an issue, but less so than the number of potential causal SNPs. We have included the following text in our discussion explaining this (with new text in red and old text in black, included for context):

> Finally, stochastic search could be used to speed up MsCAVIAR in cases where there are possibly many causal variants [10, 30]. MsCAVIAR's runtime is largely determined by the number of SNPs in the locus and the number of causal SNPs allowed: if there are M total SNPs and up to K are allowed to be causal, then there are potentially up to $M \choose K$ causal status vectors to evaluate. Thus, runtime can become an issue when there are many SNPs in a locus or many studies, and especially when users desire to allow for more than three possibly causal SNPs at a locus. Stochastic search can help reduce the search space by not evaluating every possible combination of causal SNPs, though this involves managing the risk of missing the optimally minimal causal set.

Reviewer 2 Remarks

Reviewer #2: LaPierre and colleagues present a novel approach for fine-mapping loci using summary statistics from multiple studies whilst accounting for heterogeneity in the effects of causal variants between them. The methodology can allow for multiple causal variants at a locus, and can be applied in the context of trans-ethnic fine-mapping by allowing for study-specific patterns of LD between variants. The methodology tackles an important challenge in human genetics, is extremely timely, and likely to be of great interest to the readership of PLoS Genetics. I am looking forward to trying the software! The manuscript is generally well written, although some additional details of the simulation study and the applications to trans-ethnic GWAS of type 2 diabetes and cholesterol would be useful.

Reviewer 2, Specific Comment #1 on Methodology

Comments on methodology

1. Presumably it would be straightforward to incorporate a non-uniform prior of causality? Given the availability of enrichment in associations in specific annotations, it would be really useful to allow this flexibility in the method/software.

Authors' Response

We thank the reviewer for raising this issue. MsCAVIAR can, in principle, incorporate functional priors by allowing the prior probabilities of the SNPs being causal to be different, as discussed in CAVIAR; currently, as you can see, they are all set to a fixed "gamma" prior probability. However, we are concerned that setting these priors arbitrarily is dangerous, and that more work would be needed to determine how best to model various functional priors in the context of MsCAVIAR's model. We think that this could potentially be a good direction for future research. Thus, we have included the following in our discussion:

> Functional information can in principle be factored into MsCAVIAR's model by modifying the prior distribution P(C) so that not every variant has the same prior probability of being causal, as described in the CAVIAR paper \cite{caviar}. However, setting these priors arbitrarily can yield misleading results, and future work is needed to determine how best to model various functional priors in the context of MsCAVIAR's model.

Reviewer 2, Specific Comment #2 on Methodology

2. In the context of trans-ethnic meta-analysis, could the authors provide some guidance as to whether each study should be included separately in the meta-analysis, or whether a fixed-effects meta-analysis of each ethnic group should undertaken first, and then used as input

to msCAVIAR? Presumably this would be computationally more demanding, but are there advantages in allowing for heterogeneity between studies from the same ancestry?

Authors' Response

We thank the reviewer for raising this question. We agree with your later feedback on the discussion suggesting that the strategy of performing a meta-analysis on the studies from each separate population group before applying MsCAVIAR is superior to retaining only one study from each population. We would recommend this over the approach of simply including every study in the MsCAVIAR run because, as stated in the discussion, this would not fit MsCAVIAR's model of equal heterogeneity between studies. We have changed the text to reflect this recommendation. The text now reads (see discussion, third paragraph):

> We also assume that all studies are drawn with equal heterogeneity τ^2. This is unlikely to be true if multiple studies are from a single population while another study is from a different population. In such a scenario, we recommend grouping the studies by population, running fixed effects meta-analysis on each group, and then running MsCAVIAR on the results for the different groups. Concretely, the input summary statistics for MsCAVIAR should be the results from the meta-analysis of each population, and the input LD matrices should be derived from either the genotype data (if available) or the appropriate reference panels for each population.

Reviewer 2, Specific Comment #3 on Methodology

3. Heterogeneity is modelled under a random-effects model, but would it be feasible (and beneficial) to consider alternative models (such as less heterogeneity between more genetically similar studies)? Could the tau2 parameter be considered as a hyperparameter to be estimated, and could this give some intuition as to the extent of heterogeneity?

Authors' Response

We thank the reviewer for raising this issue. Tau^2 can in principle be estimated, but it is not trivial to do so because one risks overfitting this parameter, since heterogeneity of different causal SNPs can vary across loci and an overfit Tau^2 could miss some of those SNPs. We do think that a good estimation procedure could be a good future direction to explore, however. We have added the following to the Discussion section:

> In practice, we set the τ^2 parameter to a fixed value, which was chosen to give power to detect both small and large amounts of heterogeneity (Methods, ``Parameter Setting in Practice''). This value could, in principle, be adjusted based on the apparent heterogeneity present in the data. However, care would have to be taken to not overfit the parameter to the summary statistics in each locus, since the heterogeneity of different causal SNPs can vary across

> loci and some causal SNPs may be missed when the heterogeneity parameter is overfitted. Future work could develop a procedure for fitting this parameter.

Reviewer 2, Specific Comment #4 on Methodology

> *4. It wasn't totally clear to me whether the (maximum) number of causal variants needs to be specified in advance.*

Authors' Response

We thank the reviewer for pointing this out, and we have now included this information in our new "Overview of the MsCAVIAR Model" (first subsection in Methods):

> In practice, we limit the search space $\mathcal{C}$ by allowing the user to set the maximum number of causal SNPs allowed, 3 by default.

Reviewer 2, Specific Comment #5 on Methodology

> *5. Some details of computational efficiency would be beneficial. In simulations and data applications, SNPs are thinned by various criteria. Is this because the methodology/software does not work well if there are SNPs in strong LD, or is computationally demanding if the number of SNPs is large?*

Authors' Response

We thank the reviewer for raising this question. We no longer prune SNPs in perfect LD, due to the concerns raised by multiple reviewers. But the reviewer is correct that we did this originally for computational reasons. That is also the reason for thinning SNPs with low signal -- there are often many insignificant SNPs that carry very little information and only serve to slow down computation. We discuss this further below in response to comment 4 on the Real Data Applications.

The computational complexity can't be captured easily in a single big-O expression because it also depends a great deal on the locus itself -- how many SNPs there are, the LD structure, and how many causal SNPs there actually are -- as well as the parameters supplied by the user, chiefly the maximum number of causal SNPs allowed and the posterior probability threshold for termination. However, we agree with the reviewer that adding some context is helpful.

The chief determinant of the runtime is the number of SNPs in the locus and (especially) the maximum number of causal SNPs allowed, because, if there are M SNPs and up to K may be causal, there are potentially up to M choose K causal status vectors to evaluate. We have included the following text in our discussion explaining this (with new text in red and old text in black, included for context):

Finally, stochastic search could be used to speed up MsCAVIAR in cases where there are possibly many causal variants [10, 30]. MsCAVIAR's runtime is largely determined by the number of SNPs in the locus and the number of causal SNPs allowed: if there are M total SNPs and up to K are allowed to be causal, then there are potentially up to $M \choose K$ causal status vectors to evaluate. Thus, runtime can become an issue when there are many SNPs in a locus or many studies, and especially when users desire to allow for more than three possibly causal SNPs at a locus. Stochastic search can help reduce the search space by not evaluating every possible combination of causal SNPs, though this involves managing the risk of missing the optimally minimal causal set.

Reviewer 2, Specific Comment #6 on Methodology

6. One of the nice features about Susie is that it effectively gives a credible set for each causal variant (reflecting the fact that these each represent a distinct association signal). If we were then keen to colocalise association signals with eQTLs, this could be done for each credible set separately. However, for msCAVIAR, it does not seem that we could extract equivalent information. For example, if there were two signals at a locus (and two causal variants), the first signal might be easy to fine-map, so that we are clear of the causal variant for that signal, but for the other signal, there might be several variants with equivalent fine-mapping support. Is there anyway to distinguish the fact that the variants in the credible set are somehow grouped by distinct associations, and if not, do the authors view this as a disadvantage?

Authors' Response

We thank the reviewer for raising this issue. We view the approaches of MsCAVIAR and SuSiE as having different advantages: the advantage of the former is its completeness (in terms of returning all causal signals), while the advantage of the latter (as you said) is its ability to separate out distinct signals. We think it is possible to accomplish both aims by generating a MsCAVIAR causal set and then partitioning this set into subsets with separate signals, but we view this as a substantial extension for a separate paper. We have included the following in our discussion section:

MsCAVIAR aims to return a causal set that contains all causal SNPs in a locus, while SuSiE aims to return one or more credible sets that each contain at least one causal SNP. The advantage of the former approach is its completeness in terms of identifying all causal signals, while the advantage of the latter approach is its ability to separate distinct causal signals within a locus into separate sets. A future extension to MsCAVIAR could aim to accomplish the benefits of both by returning a causal set with all causal SNPs, and then partitioning this set into distinct subsets with separate causal signals.

Reviewer 2, Specific Comment #1 on the Simulation Study

Comments on the simulation study

1. Details of the simulation study are rather scant. How large are the two regions (physical distance and number of SNPs)? I can understand removing SNPs in perfect LD to select causal variants (although in practice, I guess two causal variants could be in perfect LD), but then this only leaves 48/38 SNPs, which does not seem like a "realistic" fine-mapping scenario. I also was not clear if the SNPs included in the analysis were also LD pruned, or if all SNPs in the region were considered as potentially causal. Could there every be a situation where a causal variant was specific to just one population (i.e. monomorphic in the other) – or did causal variants have to be present in both populations (at some frequency)?

Authors' Response

We thank the reviewer for this feedback. We have revamped our simulations following the comments of reviewer 3, and now the loci are much larger (126-154 SNPs) and are not LD pruned. The causal variants are simulated to be the same across all populations, as many methods (including MsCAVIAR) assume this. Below is our updated text describing our simulations:

> We now describe our simulation study to evaluate the performance of MsCAVIAR as compared with other methods. We selected two samples of 9,000 unrelated individuals from the UK Biobank, one with European ancestry and the other with Asian ancestry. In order to generate realistic fine mapping scenarios, we centered 100kbp windows around SNPs that reached genome-wide significant association with High-Density Lipoprotein cholesterol in the UK Biobank summary statistics released by the Neale lab for White British individuals. Among these windows, we selected three loci that reflected high, medium, and low patterns of LD as defined by the proportion of SNPs with at least 90% LD (32%, 25%, and 8%, respectively). We then obtained the imputed genotype data for the three selected loci for the individuals we sampled from the UK Biobank. The loci were filtered for missing genotypes (> 0%) and low minor allele frequency (< 1%). No Linkage Disequilibrium (LD) pruning was performed. The loci with low, medium, and high LD had 154, 126, and 144 SNPs, respectively.

Reviewer 2, Specific Comment #2 on the Simulation Study

2. What is Sigma_i on line 118? It might be described later, but it is not clear what it is at this point in the manuscript.

Authors' Response

To make this clearer, we have changed the line as follows (new text in red):

We then drew the non-centrality parameter Λ_i for each study i according to $\Lambda_i \sim \mathcal{N}(\Lambda, 0.5)$, and subsequently the summary statistics

S_i for each study i according to $S_i \sim \mathcal{N}(\Lambda_i\Sigma_i,\Sigma_i)$.

Reviewer 2, Specific Comment #3 on the Simulation Study

3. The authors suggest that the improved performance of msCAVIAR over PAINTOR could be because of the modelling of heterogeneity. Could the authors investigate this further by simulating effects of causal SNPs that are homogenous across studies?

Authors' Response

We thank the reviewer for this feedback. We performed these experiments and included them in a supplementary figure, reproduced below along with the relevant text. We did not find that our results were substantially different when the effects were homogenous across studies; thus, we have acknowledged this in the text and removed the line speculating that the difference between MsCAVIAR and PAINTOR could be due to modeling heterogeneity. We believe that a fuller slate of experiments varying both MsCAVIAR's modeled heterogeneity and the actual simulated heterogeneity would be needed to fully determine the impact of modeling heterogeneity, but this would constitute a substantial amount of additional work.

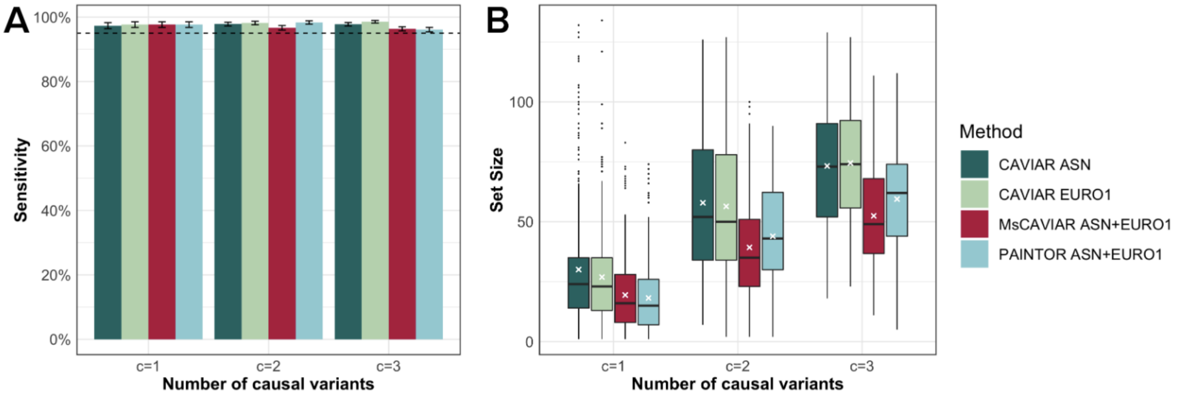

Fig. 3. Comparison of sensitivity and set sizes using simulated data with equal effect sizes. We simulated $c \in \{1, 2, 3\}$ causal variants averaging over 20 replications of 3 loci with 5 levels of heritability each. (A) Bar graph displaying the sensitivity of the methods. The dashed line reflects the expected posterior probability of recovering all causal SNPs; methods that reach this threshold are considered "well-calibrated". (B) Box plots showing the set sizes returned by the methods.

We ran simulations in which the causal effect sizes of the SNPs were equal across populations, in order to investigate whether lack of effect size heterogeneity impacted the results (Figure 3).

…

When we performed our original simulations except with effect sizes fixed across populations (e.g. no heterogeneity), the results were fairly similar to our main text results. This indicates that, in our simulations, MsCAVIAR's improved performance relative to PAINTOR is not mostly due to explicit modeling of heterogeneity.

Reviewer 2, Specific Comment #4 on the Simulation Study

4. Line 151. The authors state that the fact that msCAVIAR performs better than CAVIAR applied to each population is an indication of the improved fine-mapping resolution offered by trans-ethnic meta-analysis. However, could this actually be a reflection of the larger total ample size used by msCAVIAR? Could the authors also run simulations where the sample size of the population-specific studies are the same as a trans-ethnic study (i.e. just simulate two European studies of equal size, and compare with one European and one East Asian study of equal size)?

Authors' Response

We thank the reviewer for pointing out this possibility. Under our new simulation framework, we have performed an experiment to test this. We ran CAVIAR on a combined GWAS on two European studies of equal size and compared that with MsCAVIAR on the first European and the Asian populations. While MsCAVIAR's causal set sizes were generally slightly smaller, the results were fairly close, suggesting that sample size does largely drive the difference between CAVIAR and MsCAVIAR in our simulations -- though we caution that we don't believe that this will always be the case in reality. We also ran MsCAVIAR on the two European populations, achieving similar results to MsCAVIAR run on the two different ethnic groups. The results are summarized in the following Supplementary Figure:

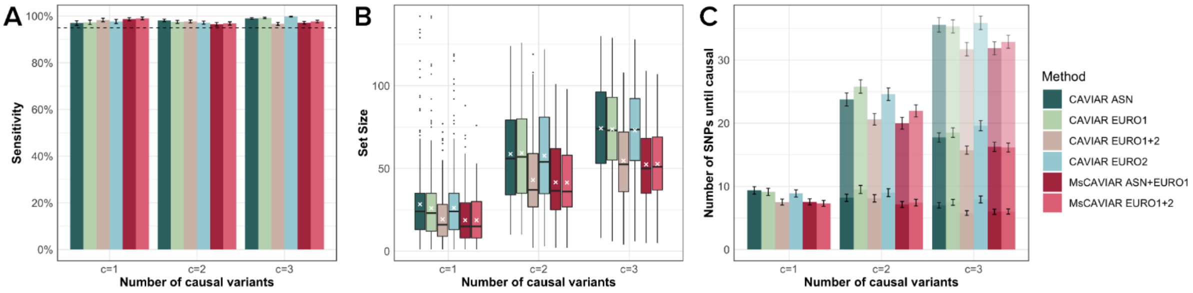

Fig. 1. Comparison of the impact of effective sample size increase to modeling heterogeneity. We simulate summary statistics with c=1, c=2, or c=3 causal variants implanted in 3 loci and 5 heritability levels with 20 replicates each. This is done for 2 different European populations and one Asian sample, all with 9,000 individuals in order to compare the impact leveraging differing LD to the effective sample size increase of meta-analysis. (A) Bar graph displaying the sensitivity of the methods (B) Box plots showing the set sizes returned by the methods. The lines inside the boxes represent the median while the white crosses inside the boxes represent the mean. (C) Bar graph showing the average number of SNPs taken in descending order of posterior inclusion probability (PIP) until 1, 2, or 3 causal SNPs are identified. Stacked bars represent increasing numbers of causal SNPs identified, until the true number of causal SNPs (x-axis) are identified.

Reviewer 2, Specific Comment #1 on the Real Data Applications

> *Comments on data applications*
>
> *1. Effective sample size is a more useful way of representing the sample size for a disease phenotype – would actually better to give the number of diabetes cases and controls for each study.*

Authors' Response

We thank the reviewer for this valid point. Following the reviewer's third comment on the real data application (see below), we expanded the locus sizes from 100kb to 1 Mb. After doing this, we had only two remaining loci for Type 2 Diabetes. For this reason, and the fact that the High Density Lipoprotein (HDL) analysis uses similar data and has many more loci, we chose to no longer include the Type 2 Diabetes analysis in the results.

Reviewer 2, Specific Comment #2 on the Real Data Applications

> *2. It would be good to give information on the loci used in comparisons in supplementary information (for both applications), together with the numbers of credible causal variants for each locus with the different methods.*

Authors' Response

We have now included a supplementary table which has this information.

Reviewer 2, Specific Comment #3 on the Real Data Applications

> *3. Centering the loci 50kb up- and down-stream of the lead SNP seems rather restrictive – we know that LD often extends over greater distances, and I think using 500kb up- and down-stream would be much more realistic. Was this done for computational reasons?*

Authors' Response

We thank the reviewer for pointing this out. We have changed the real data loci to include 500kb up- and down-stream, following this suggestion.

Reviewer 2, Specific Comment #4 on the Real Data Applications

> *4. I didn't follow the motivation for removing SNPs with p>0.0001. There could be examples where a causal variant does not have strong association in a single SNP analysis, and is only*

revealed when considering multiple causal variants (depending on patterns of LD between causal SNPs and directions of effect on the outcome). Was this done for computational reasons?

Authors' Response

The reviewer is correct that this is done for computational reasons. However, we acknowledge the point that some of the filtered SNPs may have an impact on the fine mapping analysis. We relaxed the filter to remove SNPs that did not reach even marginal significance (p > 0.05) -- we think that these SNPs are even more likely to not impact the fine mapping analysis, and including them greatly slows down the methods. The relevant updated text reads as follows:

> To generate loci for fine mapping, we centered 1 megabase windows around genome wide-significant peak SNPs (p-value <= 5*10^-8), discarding all SNPs that did not reach even marginal significance (p > 0.05), as they were highly unlikely to be informative and would slow down analysis.

Reviewer 2, Specific Comment #5 on the Real Data Applications

> *5. Figures 4 and 5. I did not find the violin plots useful in Figure 4. I understand that it is hard to summarise results when there are just five loci in Figure 4. It would be useful to have three scatter plots where the x-axis was the credible set size in msCAVIAR and the y-axis was the credible set size in one of each of the three other methods (i.e. each point is a locus) – this will provide useful information on within locus comparisons that could not be assessed with the current presentation. The box and whisker plots in Figure 5 are more useful, but I think could also be presented alongside scatter plots as described above.*

Authors' Response

We thank the reviewer for suggesting this additional graph for visualizing individual locus differences. We have added this graph to the results section; for convenience, we show it below along with the relevant text. The graph illustrates that MsCAVIAR's causal set sizes are consistently smaller than those of CAVIAR. MsCAVIAR's causal set sizes are smaller most of the time than PAINTOR's, and MsCAVIAR has a smaller median set size (31 vs 34), but MsCAVIAR's average set size is slightly higher than PAINTOR's (50.8 vs. 49.3) because of some loci where its causal set sizes are much larger than PAINTOR's.

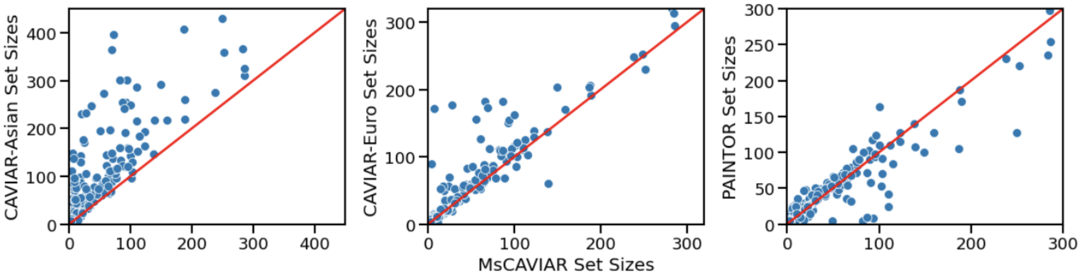

Fig. 4. Comparison of methods' set sizes for each locus in the trans-ethnic HDL analysis. Comparison of the returned causal set sizes of MsCAVIAR when applied to two high-density lipoprotein (HDL) GWAS, White European people from the UK Biobank [23, 24] and Japanese people from Biobank Japan [27, 28], versus trans-ethnic PAINTOR [15] and applying CAVIAR [8] to each population individually. In each scatter plot, each point reflects a specific locus, and the x-coordinate is MsCAVIAR's returned causal set size, while the y-coordinate is a different method's causal set size. Diagonal lines representing equal set sizes were plotted for each scatter plot. Points above the line represent loci where the alternate method had a larger causal set size than MsCAVIAR, while points below the line indicate the opposite.

As an additional way of viewing the results, we generated scatter plots of the causal set sizes at each locus for MsCAVIAR compared to those of PAINTOR and CAVIAR (Figure 4). This visualizes the comparative causal set sizes at individual loci. The scatter plots and their associated lines of equality reveal that MsCAVIAR's set sizes were consistently smaller than CAVIAR's across almost all loci, with one notable exception in which CAVIAR's causal set size was substantially smaller than MsCAVIAR's. The comparison with PAINTOR illustrates how MsCAVIAR's median causal set size was smaller than PAINTOR's but its average was higher: MsCAVIAR returned slightly smaller causal set sizes than PAINTOR for most loci, but in some cases, MsCAVIAR's causal set size was much larger than PAINTOR's, dragging MsCAVIAR's average causal set size above that of PAINTOR.

Reviewer 2, Specific Comment #6 on the Real Data Applications

6. I wasn't totally clear about the final sentence of the last paragraph (line 259). In particular, I wasn't clear about why it mattered that msCAVIAR models heterogeneity as being the same at each locus. As far as I am aware, PAINTOR also models heterogeneity as being the same at each locus (i.e. fixed effects, so no heterogeneity). So do these results imply that msCAVIAR does not perform well if effects are homogenous across studies? I think this emphasizes the importance of running some simulations under a model in which effects are the same in the two studies.

Authors' Response

We thank the reviewer for this feedback and acknowledge that the sentence in question was too speculative and perhaps not the reason for the observed results, so we have removed the sentence in question. As discussed above, we have performed the experiment with the same effects in both studies.

Reviewer 2, Specific Comment #1 on the Discussion

> *Comments on the discussion*
>
> *1. I think it would be beneficial to expand somewhat on the comment about "equal heterogeneity" – presumably this is just the underlying assumption of a random effects model? I agree that this model is not appropriate when several studies are from one population, and one study is from another (because less heterogeneity would be expected between studies from the same population). However, I disagree with the recommendation to use a single study from each population. It would be much better to meta-analyse studies from the same population/ethnicity together, and use those as input to msCAVIAR (assuming you can use the same LD matrix for all studies from the sample population).*

Authors' Response

We thank the reviewer for raising this issue to our attention. We have changed the text to reflect this recommendation. The text now reads (see discussion, third paragraph):

> We also assume that all studies are drawn with equal heterogeneity τ^2. This is unlikely to be true if multiple studies are from a single population while another study is from a different population. In such a scenario, we recommend grouping the studies by population, running fixed effects meta-analysis on each group, and then running MsCAVIAR on the results for the different groups. Concretely, the input summary statistics for MsCAVIAR should be the results from the meta-analysis of each population, and the input LD matrices should be derived from either the genotype data (if available) or the appropriate reference panels for each population.

Reviewer 3 Remarks

Reviewer #3: Review of LaPierre et al

The paper presents an extension of the fine-mapping

method (CAVIAR) to deal with multiple studies, allowing

for heterogeneity in effects among studies.

The paper uses simulation to show that this extension

(MsCAVIAR) produces better localization, in that

it reduces the size of the "causal set" produced compared with CAVIAR

(and other methods) run on the individual studies.

Results on real data show similar trends (smaller causal sets.)

This is a potentially useful -- if conceptually fairly straightforward --

extension of the CAVIAR method. However, there are several important

issues that would need addressing to make it suitable for publication.

Reviewer 3, Main Issue #1, First Part, First Paragraph

Main Issues

1. While the main text is very clearly written and nicely presented,

the Methods section is confusing and difficult to follow.

I suggest the following:

First, there needs to be a very simple and clear statement of the model and

prior distribution used. This should be separated from any "derivation" of this

model (which can likely be mostly justified by appropriate citations of previous work)

and also separated from the computational tricks (which also seem like straightforward extensions of previous work).

Authors' Response

We agree with the reviewer that an overview of the model could improve clarity. Thus, we have added a new subsection at the beginning of the Methods section, titled "Overview of the MsCAVIAR Model", which is omitted from this document for brevity. We hope that this overview helps clarify the model.

Reviewer 3, Main Issue #1, First Part, Second Paragraph

At the moment the model is very hard to extract from the text. Lambda is used in different places in different ways: at the top of p11 lambda_i = beta_i sqrt(n_i)/sigma_e , but then later in the same paragraph it is used for the mean of S, which is not the same thing. Maybe because of this there appear to be circular definitions (Lambda_C is defined in terms of Lambda at (2), and then Lambda is defined as \Sigma Lambda_C at the top of p15). [I actually don't think you need both Lambda and Lamba_C: you can just use Lamdba for the true non-centrality parameters (which will be 0 for non-causal SNPs) and then directly use \Sigma Lambda for the expectation of S, so

S| Lambda \sim N (\Sigma Lambda, \Sigma)

or something like this?]

Authors' Response

We thank the author for pointing out the "circular definition" issue and lack of clarity around Lambda and Lambda_C. We put a lot of thought into trying to remove Lambda and rename Lambda_C to Lambda, but this caused a lot of downstream notation issues that were insurmountable, so we kept

both. However, we agree with the reviewer that we should avoid a circular definition and define S | Lambda differently. We have rewritten the relevant portion of the methods section to reflect this:

> We now model the observed summary statistics $S = [s_1, \ldots, s_m]$ according to
>
> $$ S | \Lambda_C ~ N(\Sigma\Lambda_C, \Sigma) $$
>
> where Σ represents the pairwise Pearson correlations between the genotypes. $\Lambda_C = [\lambda_{C_1} ... \lambda_{C_M}]$ represents the true standardized causal effect sizes of each SNP, where each entry $\lambda_{C_m}=0$ if SNP m is non-causal and $\lambda_{C_m} \neq 0$ otherwise.
>
> …
>
> We use the shorthand $\Lambda = \Sigma\Lambda_C$ to refer to the non-centrality parameters (NCPs) of the statistics of all SNPs, which are induced by Linkage Disequilibrium (LD) with the causal SNPs. Thus, $S | \Lambda ~ N(\Lambda, \Sigma)$.

Reviewer 3, Main Issue #1, First Part, Third Paragraph

> *The extension to different sample sizes is described*
>
> *imprecisely in words (top of p23), and needs equations to make it precise.*
>
> *I would suggest just giving the model for different sample sizes*
>
> *directly, since the case where they are the same are then a special case.*
>
> *The model seems to be a "matrix normal" model, and making that explicit could help.*

Authors' Response

We agree that the different sample sizes subsection could have more precision. Because the explanation requires a substantial amount of new notation, we do feel that it is better to introduce separately, so we have left it in its own subsection. However, we have tried to adjust the text to make the section more precise. For example, in the opening paragraph for the subsection, we have removed references to the "one true non-centrality parameter" and instead set up the section as such:

> In ``Fine mapping across multiple studies'', we discussed the MsCAVIAR model, in which the non-centrality parameters $\lambda_{C_{mq}}$ for SNP m in each study q are drawn around a global mean non-centrality parameter $\lambda_{C_m} ~ N(0,\sigma^2)$ with variance

τ^2, such that $\lambda_{C_{mq}} ~ N(\lambda_{C_m},\tau^2)$. We note that λ_{C_m} is itself a function of the non-standardized effect size β_m, where $\lambda_{C_m} = (\beta_m \sqrt{N}) / (\sigma_e)$ and $\beta_m ~ N(0, \sigma^2_g)$. Thus, λ_{C_m} and its variance σ are functions of the sample size N. Since the sample size may not be consistent across the studies, this λ_{C_m} is an oversimplification that cannot be used when different studies have different sample sizes. Below, we show how to model the $\lambda_{C_{mq}}$ for each study while taking into account possibly different sample sizes.

Reviewer 3, Main Issue #1, Second Part

Second, the definitions of the summary data S need to be made clear. At the moment they are defined as beta-hat_i \sqrt(n_i)/sigma_e but sigma_e is unknown. And at line 444 you say "we now operate... that sigma_e has been standardized (sigma_e=1)". But there is no way to standardize to ensure sigma_e=1 because we do not know the true residual variance. It is common to standardize y to have unit variance, but this does not imply the residual variance is 1. (I think maybe in the model you are assuming y has been standardized to have variance 1, and then making the approximation that sigma_e \approx 1 under the assumption of low heritability? But not sure whether you are also doing this for s_i, so taking s_i = beta-hat_i \sqrt(n_i) ? Or using an estimate of the residual variance? In any cae these kinds of details, assumptions and approximations need to be more precise.)

Authors' Response

We thank the reviewer for raising this point. You are correct that we intended to assume that y is standardized and approximating that sigma_e is 1 under the assumption of low heritability. We have clarified this by writing:

> We will now operate under the standard assumption that the trait has unit variance and variance explained by any particular SNP is small, thus $\sigma_e \approx 1$.

We now present the distribution of S differently, as discussed previously.

Reviewer 3, Main Issue #2

2. The simulation study is rather too favorable to the method, and should be made more realistic. In particular i) the simulation is performed under the assumed summary data model, rather than under a more realistic full data model (ie the regression model (1)); ii) the simulation is performed with "effect sizes" (actually, non-centrality parameter) with a narrow range centered on 5.2, which not only seems also to be used in the prior, but also seems unrealistic - it will seldom produce either small difficult-to-detect effects or very large effects, both of which are likely to occur frequently in practice; iii) the simulation is done assuming the same LD structure in both the study and the inference, whereas the real data analysis uses a panel to approximate the study LD.

It would seem easy to generate more realistic simulated data by simulating outcomes Y from the full data model (1), using real genotype data (X) an a range of beta values (eg randomly drawn from N(0,sigma^2_g) for some sigma^2_g) so that both small and big effects occur.

Authors' Response

We thank the reviewer for this feedback. We have redone the simulations according to these suggestions. We used real UK Biobank genotypes, randomly implanted causal SNPs, and performed GCTA phenotype simulations under a variety of heritability levels to generate a range of effect sizes. We then performed GWAS with fastGWA to generate the summary statistics. This procedure produced 900 total simulations with peak SNP Z-scores ranging from about 4 to about 20. We have included our paper's description of our new simulations below. As our figure for the next Main Issue shows, MsCAVIAR's relative performance versus the other methods under these new simulations was similar to our previous findings.

We now describe our simulation study to evaluate the performance of MsCAVIAR as compared with other methods. We selected two samples of 9,000 unrelated individuals from the UK Biobank, one with European ancestry and the other with Asian ancestry. In order to generate realistic fine mapping scenarios, we centered 100kbp windows around SNPs that reached genome-wide significant association with High-Density Lipoprotein cholesterol in the UK Biobank summary statistics released by the Neale lab for White British individuals. Among these windows, we selected three loci that reflected high, medium, and low patterns of LD as defined by the proportion of SNPs with at least 90% LD (32%, 25%, and 8%, respectively). We then obtained the imputed genotype data for the three selected loci for the individuals we sampled from the UK Biobank. The loci were filtered for missing genotypes (> 0%) and low minor allele frequency (< 1%). No Linkage Disequilibrium (LD) pruning was performed. The loci with low, medium, and high LD had 154, 126, and 144 SNPs, respectively.

We then simulated causal SNPs and their effect sizes Beta ~ N(5.2 / sqrt(9000), 1), for the cases of 1, 2, or 3 causal SNPs randomly chosen within each locus. For simplicity, we take the absolute value of the effect size and restrict causal SNPs to being positively correlated with each other. We then used GCTA to simulate phenotypes using different heritability levels: 0.2%, 0.4%, 0.6%, 0.8%, and 1%, times the number of causal SNPs. Concretely, GCTA simulates the phenotypes y according to $y = X\beta + e$, where X is the standardized genotype matrix for the causal variant(s), β is the vector of causal variant effect sizes, and e is a vector of environmental noise terms where each $e_i = \sigma^2_g (1 / h^2 - 1)$. In other words, the environmental variance is scaled to achieve the desired heritability. Thus, modulating the heritability affects the strength of the association signal between variants and the phenotype, while drawing different β_i for different causal variants allows for the modeling of heterogeneity.

Finally, we run a linear regression using fastGWA to generate the summary statistics. We simulated 20 replicates (re-drawing the causal SNPs and their effect sizes) for each level of heritability and number of causal SNPs for a total of 900 simulations.

Reviewer 3, Main Issue #3

*3. As argued in Wang et al, the idea of outputting a "causal set" that, with high probability, contains *all* causal SNPs is flawed. There are two reasons for this. First, it can ignore a lot of useful information. For example, suppose there are two causal SNPs, and that one is in LD with just itself, but the other is in LD with 50 others. Then the causal set will*

contain (at least) 52 SNPs, and does not include the information that one SNP is very precisely mapped (which a user could clearly find helpful!)

Second, if we allow that SNPs may have small effects, which is realistic, then it becomes impossible for any method to be confident to include all the causal SNPs in a set (at least, not without the set being very large). The paper sidesteps this issue by avoiding simulations with small effects, which should be rectified (see 2 above).

In light of this it seems unsatisfactory to rely on the size of the causal set as the only indicator of improved performance, and I think the paper should also provide other evidence for the superiority of the multi-study approach. One possibility would be to demonstrate the benefits in terms of PIPs (posterior inclusion probabilities). For example, does MsCAVIAR show a better precision-recall curve (equivalently, true-positive rate vs false discovery rate) as PIP threshold is modified?

Authors' Response

We thank the reviewer for this insightful point. Since the number of SNPs varies across loci, we felt that a precision-recall curve averaged across loci may not be the best representation of the results. We tried to achieve what the reviewer suggests by constructing a graph showing the number of SNPs taken in descending PIP order until each causal SNP is selected. This captures the idea of how well SNPs are actually prioritized/ordered, in a set-agnostic way. We show the graph for this below, which is subfigure C in our overall simulation results figure. This figure shows that, not only were MsCAVIAR's average and median set sizes smaller than PAINTOR's when there were multiple causal SNPs, but its ordering of SNPs by PIP was also slightly superior.

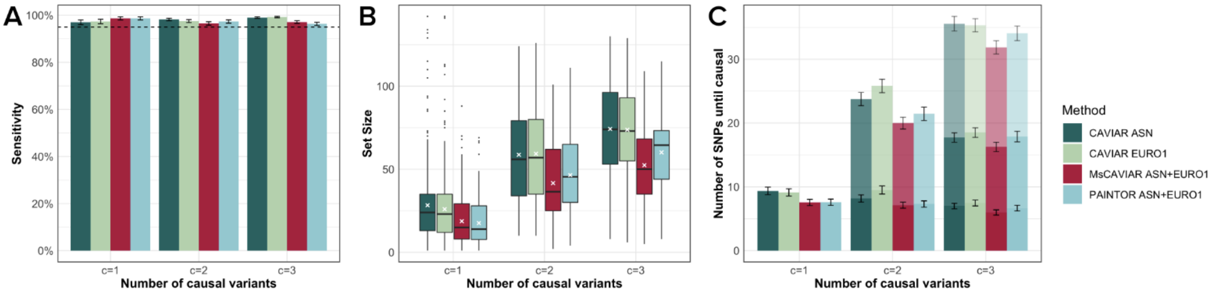

Fig. 2. Comparison of sensitivity, precision, and set sizes using simulated data. We compare MsCAVIAR, PAINTOR, and CAVIAR with $c \in \{1, 2, 3\}$ causal variants implanted with results averaged over 20 replicates for 3 loci and 5 levels of heritability for all 3 values of c. (A) Bar graph indicating the sensitivity of each method with a dashed line to reflect the expected posterior probability, ρ, of recovering all causal SNPs (B) Box plots showing the average set sizes returned by the methods. Each box is the interquartile range of causal set sizes with the middle black line representing the median, and the white crosses showing the mean. (C) Bar graph displaying the average the number of SNPs in descending order of posterior inclusion probability (PIP) until 1, 2, or 3 causal SNPs is identified. Stacked bars represent increasing numbers of causal SNPs identified, until the true number of causal SNPs (x-axis) are identified.

All of the methods in this assessment were well-calibrated (Figure 2a), which is expected, as previously shown for CAVIAR \cite{caviar} and PAINTOR \cite{paintor-trans-ethnic}. For each number of causal SNPs, MsCAVIAR and PAINTOR returned substantially smaller set sizes than CAVIAR run on either population individually, highlighting the benefit of utilizing information from multiple studies.

With one causal SNP in the locus, MsCAVIAR and PAINTOR had similar causal set sizes, with MsCAVIAR's mean and median set sizes being 18.7 and 15.0 and PAINTOR's being 17.6 and 14.0, respectively. When there were two causal SNPs simulated, MsCAVIAR's causal sets were smaller on average than PAINTOR's, and the difference increased when three causal SNPs were simulated. When two causal SNPs were simulated, MsCAVIAR's mean and median set sizes were 41.6 and 36.5, respectively, while PAINTOR's mean and median set sizes were 46.4 and 45.5, respectively. Finally, with three causal SNPs, MsCAVIAR had mean and median set sizes of 52.4 and 50.0, respectively, and PAINTOR's were 60.1 and 64.5, respectively.

As the goal of most statistical fine mapping methods is to prioritize variants for functional follow-up, it lends the question of how informative a variant's posterior probability is to its causal status. We, therefore, sort the SNPs in descending order of posterior probability to determine on average how many SNPs are added to the causal set before the causal SNP(s) is placed in the causal set.

We evaluated this quantity for MsCAVIAR, PAINTOR, and CAVIAR run on the Asian and European populations (Figure 2c). MsCAVIAR and PAINTOR were generally better at prioritizing variants than CAVIAR, again highlighting the importance of utilizing multiple studies when possible. On average, MsCAVIAR was able to capture the causal variant(s) with fewer SNPs than PAINTOR.

Reviewer 3, Main Issue #4, Parts 1-2

4. The filtering in the real data analysis seems very ad hoc. and it is not clear

why it is done or whether it is necessary. Is it necessary to make the method's

performance look good compared with other methods? If so, this seems worrying.

If not, why not present results on much less filtered data? (even if it

may be more computationally intensive).

To comment in more detail on the filters:

i) Discarding SNPs with marginal

p values >0.0001 could miss signals as SNPs can become more significant

once one controls for other SNPs in LD.

ii) The logic that if the peak SNP is genome-wide

significant in one population and >0.0001 in another then the second population

won't help with localization isn't clear: first,

the potentially different patterns of LD in the two

populations mean that a second population could still help with localization even

without a genome-wide significant association; second maybe there are secondary SNPs

that will only show up as significant when one analyzes both populations.

Authors' Response

We thank the reviewer for raising this issue. The filtering is done for computational reasons, not to make MsCAVIAR's performance look better. We considered SNPs with weak p-values to be unlikely to impact the fine mapping analysis and a substantial computational burden for the fine mapping methods. However, we acknowledge the point that some of the filtered SNPs may have an impact on the fine mapping analysis with a cutoff of $p > 0.0001$. We relaxed the filter to remove SNPs that did not reach even marginal significance ($p > 0.05$) -- we think that these SNPs are even more likely to not impact the

fine mapping analysis, and including them greatly slows down the methods. The relevant updated text reads as follows:

> To generate loci for fine mapping, we centered 1 megabase windows around genome wide-significant peak SNPs (p-value <= 5*10^-8), discarding all SNPs that did not reach even marginal significance (p > 0.05), as they were highly unlikely to be informative and would slow down analysis.

Reviewer 3, Main Issue #4, Part 3

> *iii) You say "fine mapping is not as useful when there are few strongly associated SNPs".*
>
> *Why? I would think these loci may give the potential to fine map quite precisely!*
>
> *Surely the problem cases are whether there are many SNPs in strong LD, all strongly associated,*
>
> *which makes fine mapping difficult?*

Authors' Response

This statement was meant to convey that fine mapping may not be necessary when there is little doubt about what the causal SNP is. As an extreme example, if one SNP has a genome-wide significant p value and no other SNP has even a marginally significant p value, then fine mapping is not particularly helpful, as it is very likely to just claim what is already expected, that the single significant SNP is causal. In such cases, fine mapping doesn't have particularly high utility. We agree that fine mapping is easier in these cases -- in fact, perhaps too easy to even differentiate between competing methods -- but it also has less utility. In order to help clarify our view on this, we have updated the text as follows:

> We also excluded all loci with fewer than ten SNPs in each study after filtering SNPs with p>0.05, as fine mapping may not be seen as necessary or may be trivial for existing methods when there are few strongly associated SNPs.

Reviewer 3, Main Issue #4, Part 4

> *iv) "As a final step, we pruned groups of SNPs that were perfectly correlated with*
>
> *each other in both studies... would cause the LD matrix to be low rank". Does*
>
> *this mean you pruned if they were perfectly correlated in the *study* samples*
>
> *or in the LD panel? It could make sense to pool together SNPs that are perfectly*

correlated in the study, but if they are perfectly correlated in the panel

but not in the study then it seems you would want to keep them both.

(In that case perhaps you need the methods that allow for

low rank LD matrices in the panel, eg using the methods you cite

from Lozano et al.)

Authors' Response

We thank the reviewer for pointing out this distinction. We have updated our analyses to not perform LD pruning. As the reviewer expected, this required the implementation of the method from Lozano et al, which we have completed. As far as we are aware, this is a unique feature among MVN-based fine mapping methods. We added a subsection to the methods section, "Handling Low Rank LD Matrices", that briefly discusses this method and refers readers to Lozano et al. We have not included this subsection in this document, for the sake of brevity.

Reviewer 3, Other Issues #1

Other issues

- the caption to Figure 2 should include some explanation of the fact that the

calibration of SuSie can't be compared to the other methods because its sets

have a different goal.

Authors' Response

We have added the following clarification to the captions of the simulation figures:

> SuSiE's credible sets differ from the causal sets of the other methods in that SuSiE does not attempt to capture all causal SNPs, so the sensitivity calibration is not directly comparable to the other methods.

Reviewer 3, Other Issues #2

- at line 110-111, I understand you pruned SNPs in perfect LD to reduce computation

and possibly to reduce low-rank issues for MsCAVIAR. However, while pruning may initially

seem innocuous, it raises several concerns. For example, a group of 10 SNPs in complete LD should

have approximately 10 times the probability that at least one of them

is causal compared with a single SNP that is in LD only with itself.

Most analysis methods would take that into account if the SNPs were simply

in "very high LD", but this is hard to do if

you have pooled/pruned the SNPs. And the pruning will understate

the size of typical causal sets (and so overstate performance) for all methods.

Also posterior quantities of interest (eg posterior inclusion probabilities)

may be difficult to correct for this pooling. The bottom line: if

pooling is just a way to reduce computation, can you show

that results of analyzing data with pooling are similar to results

without pooling?

Authors' Response

We thank the reviewer for these insightful observations. As discussed under "Main Issue #4, Part 4", this motivated us to implement the low rank method by Lozano et al, so that we no longer have to perform LD pruning.

Reviewer 3, Other Issues #3

- line 131-3 suggest that Fig 2 boxplots are for only a subset of

the simulations (even though the Figure caption does not mention it).

That seems dangerous, and I do not see why

not to include all simulations in the boxplot.

Authors' Response

As we have changed the simulations (as described above), this comment no longer applies to our new simulations. As described above, loci with more than 200 SNPs were not fine mapped in our new simulations, but only for computational reasons. They were not evaluated at all by any of the methods,

so we did not exclude them to make MsCAVIAR look good -- we do not know whether MsCAVIAR would have been better or worse than the other methods on such loci.

Reviewer 3, Other Issues #4

> *- line 148-9; they are only equivalent under the *assumption* that there is 1 causal SNP,*
>
> *and not when methods are applied to data where the truth is only 1 causal SNP but they*
>
> *do not assume only 1 causal SNP.*

Authors' Response

Thank you for pointing out this distinction. We have changed the relevant line to:

> It is worth noting, however, that SuSiE's credible set is equivalent to the causal set (as defined by the other methods) when the methods assume that there is only one causal SNP in a locus.

Please note that this line appears only in the Appendix now, which is where we have relocated our old simulations to.

Reviewer 3, Other Issues #5

> *- line 218: Do the reported set sizes include all SNPs that were pruned*
>
> *for being in complete LD with*
>
> *selected SNPS? It seems that they should in order to give an accurate impression*
>
> *of the effectiveness of fine mapping in practice.*

Authors' Response

As previously discussed, LD pruning is no longer performed.

Reviewer 3, Other Issues #6

> *- In the real data, how many causal SNPs do you estimate/identify at each locus?*

Authors' Response

We do not estimate a specific number of causal SNPs at each locus, we simply aim to return a set that contains all causal SNPs. However, MsCAVIAR and other methods were run under the assumption of a

maximum of 3 causal SNPs per locus, for computational reasons. We added a supplementary table that shows the causal set sizes for each method at each locus.

Reviewer 3, Other Issues #7

> *- l273: this advice seems useless because how would one know? How much worse is MsCAVIAR than CAVIAR if effects are unique to one population? One might hope that it would be robust to this because of the heterogeneity in the model, and this robustness seems worth assessing in simulations.*

Authors' Response

When we wrote this, we imagined the scenario where one population has one (or more) causal SNP(s) at a locus while the other population has zero; in this case clearly one population would have no GWAS signal and it would be inappropriate to include it in a MsCAVIAR run. However, considering your comment, it is also possible that, say, one population has three causal SNPs while the other has two, and it may not be obvious from the data. Thus, we have tried to clear up this advice in the discussion. Here is the new text (changes in red, old text in black, included for context):

> It has been shown that many causal SNPs are shared across populations [12, 17, 18]. MsCAVIAR is designed to leverage this phenomenon for increased power; however, causal variants may be unique to one population. In those instances, MsCAVIAR's model doesn't match the data, so it may not be well-calibrated or it may return large causal sets. If one population has an obvious GWAS signal while the other population(s) lack even a marginally significant signal in the same locus, applying CAVIAR to the population with signal may be more appropriate.

Reviewer 3, Other Issues #8

> *- Please provide a stable link to the code used to perform the simulations and data analysis*

Authors' Response

We have created a separate GitHub repository containing the scripts used to perform the simulations and real data analysis: https://github.com/nlapier2/mscaviar_replication. We have linked to the main repository as well as this one in a new sub-section, "Code Availability", after the methods section.

Reviewer 3, Details #1

Details:

- l99: "dividing by the sum of posterior probabilities of all configurations": isn't this necessarily 1?

Authors' Response

This is not necessarily 1 because we set a limit on the number of causal SNPs allowed, so we don't sum over the causal status vectors that indicate more than that number of causal SNPs.

Reviewer 3, Details #2

- l101: "continue increasing the size of the causal set" how is this done?

Authors' Response

We thank the reviewer for pointing out the confusing language. What was meant by this is that we first evaluate all causal sets containing one SNP, then all sets with two SNPs, and so on. The text has been changed to:

> We start by assessing causal sets containing only one SNP, and then causal sets containing two SNPs, and then three SNPs, and so on until one of the causal sets exceeds the posterior probability threshold ρ.

Reviewer 3, Details #3

*- l107-8: do you mean 20%/80% of SNP *pairs*?*

Authors' Response

Yes, we have added "pairs" to the text; see lines 107 and 108 in the updated manuscript.

Reviewer 3, Details #3

- l114: don't use effect sizes and non-centrality parameter interchangeably as they are different.

Authors' Response

We have replaced both instances of "effect size" with "non-centrality parameter" -- see the second paragraph under "MsCAVIAR improves fine mapping resolution in a simulation study".

Reviewer 3, Details #4-6 and #9

> *- l115: casual -> causal*
>
> *- l153 effect size -> non-centrality parameter*
>
> *- l190: Should "White European" be "White British"?*
>
> *- l339,341 posterior predictive -> predictive*

Authors' Response

Thank you for finding these typos; we have fixed them.

Reviewer 3, Details #7

> *- In the methods section using (lower-case) sigma for a variance (eg in equation (4)*
>
> *and subsequently) is confusing. Use sigma^2 for a variance. Related to this, line 294 I think should be*
>
> *sigma_e^2, and line 448 \sigma_g should be squared.*

Authors' Response

Thank you for pointing out this issue. We have changed these and other "sigma" variances to "sigma^2" throughout the paper.

Reviewer 3, Details #8

> *- l338 "the integral above is intractable..." but then you say closed form is available!*

Authors' Response

Thank you for pointing out this confusing wording. We have changed this line (first line under "Efficient computation of likelihood functions") to:

> The integral above is intractable in the absence of parametric assumptions about the data.

Reviewer 3, Details #10

> *- l352: " rows ... are zero" isn't this only true if you set epsilon=0?*
>
> *It would be helpful to be more consistent throughout about treatment*
>
> *of epsilon and what value it takes.*

Authors' Response

Since we are implementing the low rank method described previously, we no longer use the epsilon parameter.

Reviewer 3, Details #11

> *- in equation before l438 sqrt(n_m) should be n_m?*

Authors' Response

We thank the reviewer for finding this error; we have fixed it in the new version.

Reviewer 3, Details #12

> *- line 459, say where 5.2 comes from*

Authors' Response

Thank you for drawing our attention to this. We have added the following line in the "Parameter setting in practice" section:

> This value corresponds to the traditional genome-wide significant Z-score of 5.2, for which the two-sided Wald test p-value is 5×10^{-8}, which is considered significant by (conservatively) correcting for multiple testing [35].
