# Supplementary material for: Identifying causal variants by fine mapping across multiple studies
Source: PLoS Genet. 2021 Sep 20;17(9):e1009733. doi: 10.1371/journal.pgen.1009733 (PMC8491908; doi:10.1371/journal.pgen.1009733)
Supplement: Attachment — Submitted filename: MsCaviar_Response_to_Reviewers.docx [file pgen.1009733.s003.docx]

UNIVERSITY OF CALIFORNIA, LOS ANGELES

BERKLEY	•	DAVIS	• IRVINE • LOS ANGELES • MERCED	•	SANTA BARBARA

RIVERSIDE	•	SAN DIEGO • SAN FRANCISCO	•	SANTA CRUZ

July 11th, 2021

Dear Dr. Zeggini and Dr. Balding,

Thank you for the opportunity to submit a revised version of our manuscript, “Identifying Causal Variants by Fine Mapping Across Multiple Studies,” for publication in PLoS Genetics. We thank you and the reviewers for your time and effort, and we appreciate the feedback and how it has improved our paper. We considered the reviewers’ suggestions and as a result, we have included additional simulations and clarifying edits, particularly in the methods section. Details are included below with inline responses to the reviewers, with new text in red.

We would like to thank the reviewers for their comments, as we believe that the paper and method are stronger as a result of addressing them. In brief, we have added simulations showing the impact of out-of-sample LD on fine-mapping, and added more detail to the methods overview as suggested. We would also like to thank the editors again for considering our revised manuscript.

Sincerely,

Eleazar Eskin

Professor

University of California, Los Angeles

**Response to Reviewers’ Comments**

**Reviewer 1 Remarks**

*Reviewer #1: The authors have largely addressed all of my concerns. I have no further comment.*

**Reviewer 2 Remarks**

*Reviewer #2: The authors have made extensive revisions to the manuscript and have dealt with all comments. I look forward to trying out the software!*

**Reviewer 3 Remarks**

*Reviewer #3: Reveiew of LaPierre et al (revised)*

*The authors have been very responsive to the previous reviews, and the manuscript is much improved.*

*I have just one major remaining issue, and a few remaining minor comments I would ask the authors to address.*

**Reviewer 3, Main Issue #1**

*Although the new simulation study is much improved, it still shows results only for "in sample" LD matrices.*

*As the authors note, when using summary data it is very common to use "out of sample" LD matrices (eg LD matrices from a similar population reference panel like 1000 Genomes), and indeed the real data*

*analysis used that out-of-sample strategy. It is important for readers and users to understand whether this impacts accuracy, and so the simulation study should show results for both in-sample and out-of-sample LD matrices.*

**Authors’ Response**

We thank the reviewer for raising this issue to our attention. We performed these experiments and included them in a supplementary figure, reproduced below along with the relevant text. We find that using out-of-sample LD does degrade the fine-mapping results with the degree of effect being influence by how well the out-of-sample LD reflects the in-sample LD.

The methods CAVIAR \cite{caviar}, PAINTOR \cite{paintor-trans-ethnic}, and MsCAVIAR are designed such that they only require the z-scores as summary data and may use an external LD matrix representative of the samples for fine-mapping. Previous work has shown that this approach is underpowered \cite{bayesian-multisnp}. In the results section ``MsCAVIAR improves fine mapping resolution in a simulation study" we provide each fine-mapping method the LD matrices generated in-sample. Here, we simulate data in an almost identical fashion, but we generate the input LD matrices using the 1000 Genomes Project \cite{1000genomes} to demonstrate the impact of the out-of-sample LD matrices on our fine-mapping results. We exclude SuSiE \cite{susie} from this set of analyses as the method was not designed to accept summary data.

For the results shown in Figure 8, we use two sets of 9,000 unrelated individuals from the UK Biobank with European and Asian ancestry and then identify the corresponding populations in 1000 Genomes Project to generate the out-of-sample LD matrices. For the 9,000 UK Biobank samples with European ancestry, we select the 503 samples in the super population ``EUR" in the 1000 Genomes Project as the reference sample. For the individuals with Asian ancestry, we needed to use two super populations ``SAS" and ``EAS" due to the UK Biobank sample containing 1600 individuals with Chinese ancestry, 5900 individuals with Indian ancestry, and 1800 individuals with other Asian ancestry. We generate our representative sample using all 489 ``SAS" individuals and 123 ``EAS" individuals sampled across subpopulations. This sub-sampling was done to approximate the proportion of individuals with Chinese and Indian ancestry. We note that while our example of out-of-sample LD for Asian ancestry is more extreme than when only 1 ``super population" is used, it highlights how the accuracy of the out-of-sample LD impacts fine-mapping.

We looked at the three loci with low, medium, and high LD described in ``MsCAVIAR improves fine mapping resolution in a simulation study" and retained the SNPs with MAF $> 0.05$ in the two samples generated from the 1000 Genomes Project. This resulted in 144, 125, and 149 SNPs for the low, medium, and high LD loci, respectively. Using this set of SNPs, we simulate the causal variants identically to the process described in the main results section referenced above. The only difference was that we provide the LD matrices generated from the 1000 Genomes samples to the methods instead of their in-sample LD matrices.

In the results shown in Figure 8, we compare MsCAVIAR to trans-ethnic PAINTOR \cite{paintor-trans-ethnic} and to CAVIAR \cite{caviar} run on the Asian and European populations, separately. We average the results over the set of simulations for each number of causal SNPs: 1, 2, and 3. All methods were run using $\rho^* = 0.95$ as the posterior probability threshold; therefore methods were considered ``well-calibrated" if their sensitivity was at least 95\% (dashed line in Figure 8a), and we set MsCAVIAR's heterogeneity parameter to $\tau^2 = 0.52$ (see Methods). We evaluated the precision of the methods in Figure 8b where we show the causal set size. This metric is informative when the method returns the causal variant(s) because then fewer non-causal variants or ``false positives" are being returned in the set.

When there is only 1 causal SNP, all methods are well-calibrated; however, only CAVIAR when analyzing European samples is well-calibrated when there are 3 causal SNPs and is slightly below the threshold for 2 implanted causal variants (94.3\%). All other methods see a serious degradation in their sensitivity. This decrease in performance is a result of a poor approximation to the LD matrix for individuals with Asian ancestry. When the out-of-sample LD accurately reflects the sample, as is the case for European ancestry, CAVIAR returns results comparable to when an in-sample LD matrix is provided. For MsCAVIAR and PAINTOR, however, we see incorporating two populations does not help when one sample's LD is poorly approximated. Though the set sizes are smaller in Figure 8, the specificity is also lower than either run of CAVIAR. While MsCAVIAR and PAINTOR are both poorly calibrated, we see that MsCAVIAR is more robust to the out-of-sample LD than PAINTOR. Further work would need to be done to explore this phenomenon.

At present, we encourage users to use the in-sample LD matrix whenever possible. If this is not a possibility, we advise the user to interpret their results with the understanding the out-of-sample LD may fail to provide well-calibrated results, and the quality of results depend how well the out-of-sample LD approximates the in-sample LD. Future work could also enable the method to incorporate the sufficient summary data described in \cite{bayesian-multisnp}.

**Reviewer 3, Other Issues #2**

*The MSCaviar model is essentially specified by the equation after l296, S|C \sim N(0, \Sigma + \Sigma \Sigma_C \Sigma). I suggest to number that equation, since it is key. Further, please add at this point the precise descriptions of \Sigma and \Sigma_C. \Sigma is a block-diagonal matrix (not a diagonal matrix) with each block containing the LD matrix from a study. And \Sigma_C is given in the equation after l429, which you could*

*give here. It might seem a minor point, but it took me quite a while to collect those pieces all together to convince myself the model had been fully specified. Putting them all in the same place will help the reader see the model immediately, and numbering the key equations would help future writers refer to them if necessary.*

**Authors’ Response**

We thank the reviewer for highlighting this issue. We have numbered all equations in the methods section, and correctly called \Sigma a block-diagonal matrix. We also define \Sigma_C here and include additional text to provide a better overview of the model. The relevant edits are below:

$\Sigma$ is a block-diagonal matrix where each block corresponds to one study's LD matrix and $\Sigma_C$ is a matrix modeling the covariance structure between the causal SNPs. Further computational details on the model are provided in the subsections below, but we will make two statements here for clarity. …

The second being that Equation 3 also depends on the assumption of how the causal variants in each study relate to one another. … $\Sigma_c$ can be written using the following Kronecker product ($\otimes$)

\begin{equation}

\Sigma_C = (\tau^2I_Q + \sigma^2 1_Q1_Q^T) \otimes \mbox{diag}(1_{causal})_M

\end{equation}

where $\sigma^2$ corresponds to the non-centrality parameter's variance as seen in the per-study setting (see Equation 10) which we assume is identical across studies and where $\tau^2$ captures the heterogeneity between studies (see Equation 21). Let $1_Q1_Q^T$ be a matrix of all 1s, $I_Q$ an identity matrix, and $diag(1_{causal})_M$ be an $(M \times M)$ diagonal matrix whose diagonal entries are given by the $(1 \times M)$ indicator vector $1_{causal}$ whose entries $m$ are 1 if SNP $m$ is causal and 0 otherwise. The Kronecker product for $\Sigma_C$ is stated again as Equation 26 in the section "Efficient meta-analysis" where it is more fully described.

**Reviewer 1, Other Issues #3**

*I believe it would be further useful to state here that this model comes from assuming S_q | \Lambda_q \sim N(\Sigma_q Lambda_q, \Sigma_q) independently for each q where S_q are the summary data in study q,*

*Sigma_q is the LD matrix in study q and Lambda_q is the NCP in study q. (This basic assumption is stated in words in the main text, but seems worth repeating here). This is combined with a prior on Lambda*

*vec(\Lambda) \sim N(0, \Sigma_C) where vec(\Lambda) is the KM vector of concatenated NCPs from the K different studies to give the final model.*

**Authors’ Response**

We thank the reviewer for highlighting this issue. We have included this information in the methods overview and include the text here:

The first being that the likelihood function in Equation 3 depends on the assumption that the summary data $S_q$ for each study $q$ is independently distributed as such: $S_q | \Lambda_q \sim \mathcal{N}(\Sigma_q \Lambda_q, \Sigma_q)$ where $\Sigma_q$ is the LD matrix for the study and $\Lambda_q$ is its non-centrality parameters. This is then coupled with the assumed prior for $\Lambda_q |C \sim \mathcal{N}(0, \Sigma_{C_q})$ where $\Sigma_{C_q}$ is the covariance structure between causal SNPs for study $q$. Using the distribution of $\Lambda_q$ as a conjugate prior, the overall distribution in a single study is $S_q | C \sim \mathcal{N}(0, \Sigma_q + \Sigma_q\Sigma_{C_q}\Sigma_q)$. This is restated and more fully described in the following section (``Fine mapping in a single study"), particularly in Equations 8-11.

The second being that Equation 3 also depends on the assumption of how the causal variants in each study relate to one another. We began by concatenating the non-centrality parameters across Q studies where each contains M SNPs to create the QM-length vector $vec(\Lambda)$. The distribution of $ vec(\Lambda)$ is $vec(\Lambda)

**Reviewer 1, Other Issues #4**

*In equation (10) I would assume S_q denotes the data for study q. But then this equation seems irrelevant as you want p(C* | S) [ie posterior given all the data] and not p(C* | S_q).*

**Authors’ Response**

We thank the reviewer for pointing this issue out. Equation 10, now Equation 22 is meant to show how having a true overall non-centrality parameter from which each study’s non-centrality parameter is drawn is incorporated into posterior given the data from the one study. We fixed the typos that omitted the appropriate subscripts on lines 426 and 427 and the denominator of Equation 22 specifically: $\Sum_{c \in C} P(S | \Lambda, c)$ is now $\Sum_{c \in C} P(S_q | \Lambda_q, c)$
